# Synthesis, antimicrobial evaluation, and computational investigation of new triazine-based compounds via DFT and molecular docking

**DOI:** 10.1038/s41598-025-27847-4

**Published:** 2025-11-29

**Authors:** Aisha O. Hussain, Aisha Y. Hassan, Anhar Abdel-Aziem, Eman S. Abou-Amra

**Affiliations:** Department of Chemistry, Faculty of Science (Girl’s Branch), Al-Azhar University, Yousef Abbas Street, P.O. Box 11754, Nasr City, Cairo Egypt

**Keywords:** Chemistry, Drug discovery

## Abstract

A green synthesis protocol produced triazine derivatives such as imidazo-triazine, pyrimido-triazine, pyrazolotriazine, and triazolotriazine, which were subsequently evaluated for their antimicrobial activity.

**Supplementary Information:**

The online version contains supplementary material available at 10.1038/s41598-025-27847-4.

## Introduction

**Fig. 1:**
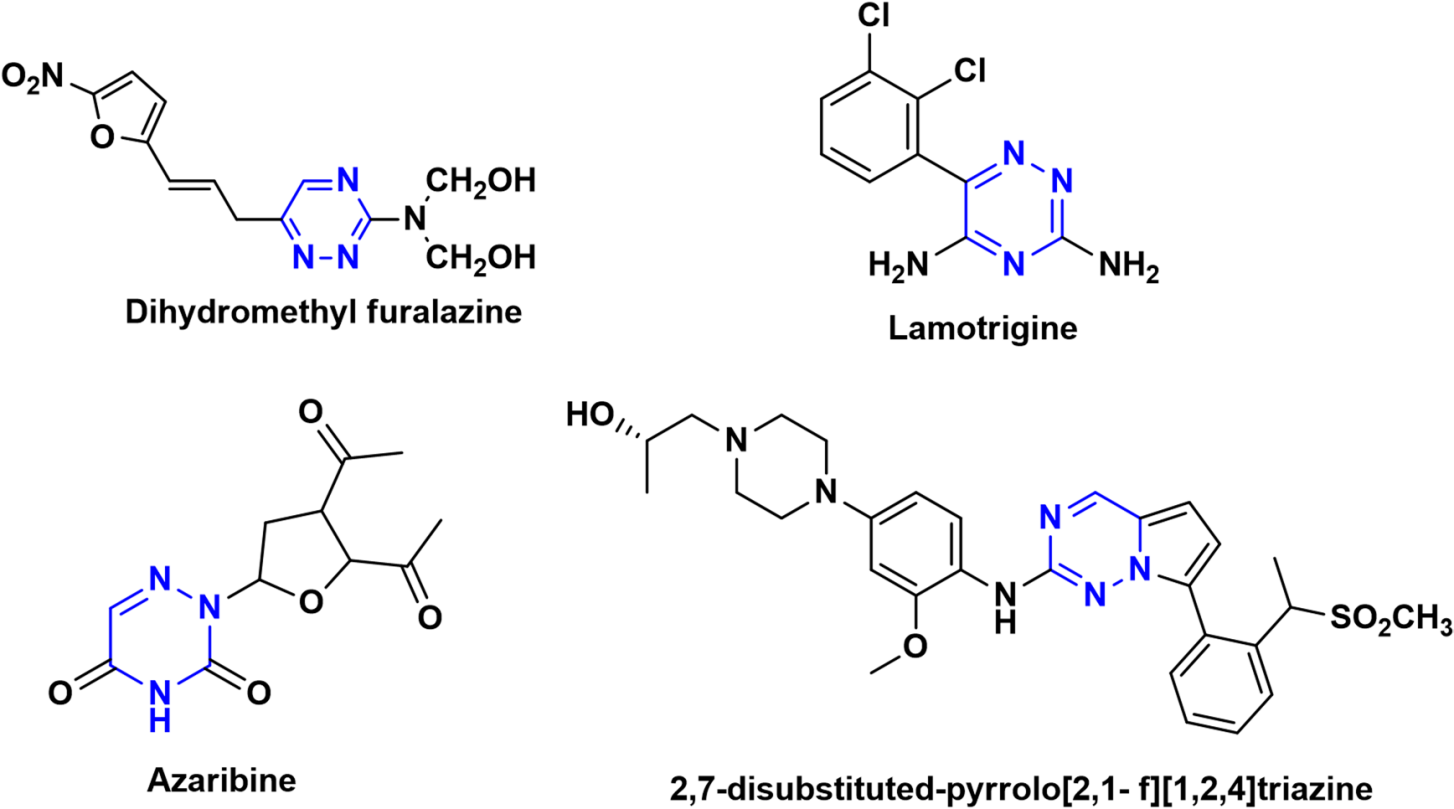
Marketed drugs containing 1,2,4-triazine scaffold.

Nitrogen-containing heterocycles, particularly 1,2,4-triazine derivatives, play a crucial role in drug discovery because of their extensive range of pharmacological actions^1^, These include antimicrobial^2^, antifungal^3^, antiproliferative^4–7^, antioxidant^8^, antiviral^9^, antiprotozoal^10^, anti-inflammatory^11^, analgesic^12^, anticancer^13–15^, anti-HIV^16^, antileishmanial^17^, cytotoxic^18,19^, and neuroleptic^20^ properties. Additional reported activities antihistaminic^21^, antimalarial^22,23^, cyclin-dependent kinase inhibition^24^, anti-tuberculosis, estrogen receptor-modulating^25^, and anti-parasite activity^26^. The structural versatility of 1,2,4-triazine derivatives and their remarkable therapeutic promise have established them as central motifs in modern medicinal chemistry^27^. Pharmaceutical agents incorporating the 1,2,4-triazine moiety exhibit diverse therapeutic effects; notable examples include lamotrigine^28^, dihydromethyl furalazine^29^, azaribine^30^, and 2,7-disubstituted derivatives of pyrrolotriazines^31,32^. (Fig. 1).

However, significant challenges persist in the creation of eco-friendly synthesis processes for novel triazine derivatives and in elucidating their structure-activity relationships (SAR) through integrated computational and experimental approaches. Conventional strategies often rely on solvent-dependent synthesis, which contradicts green chemistry principles, and lack systematic investigation of antimicrobial mechanisms using molecular modeling techniques. To address these limitations, we developed a solvent-free fusion strategy to synthesize newly 5,6-dimethyl-substituted 1,2,4-triazine scaffolds^33^ (e.g., **5**, **9**) with enhanced antimicrobial efficacy.

Our objectives are threefold: (1) to establish an environmentally sustainable synthetic route^34,35^, (2) to evaluate antibacterial and antifungal activity, and (3) to elucidate binding mechanisms via density functional theory (DFT) and molecular docking simulations targeting DNA gyrase (PDB: 4KTN) and CYP51 (PDB: 4WMZ). This study combines synthetic innovation with computational insights to advance triazine-based antimicrobial agents against drug-resistant pathogens.

## Results and discussion

**Fig. 2:**
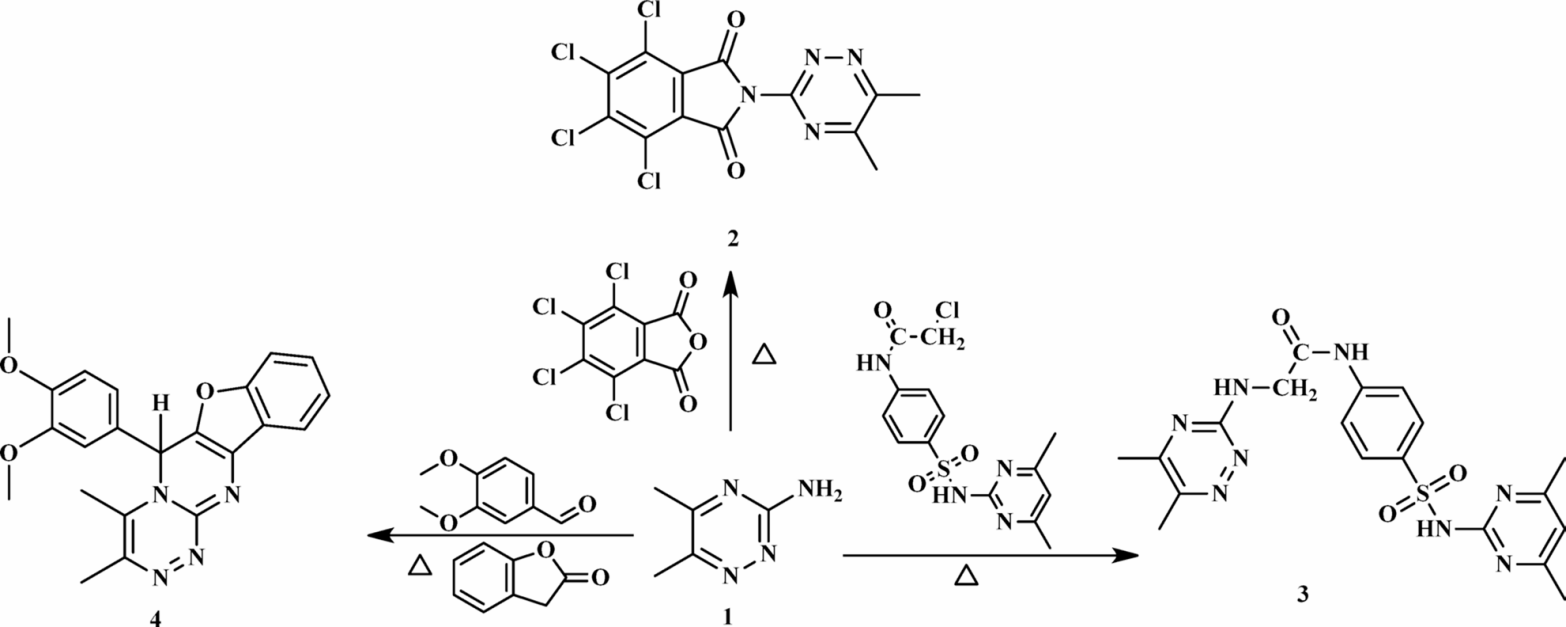
Synthesis of compounds (2–4).

**Fig. 3:**
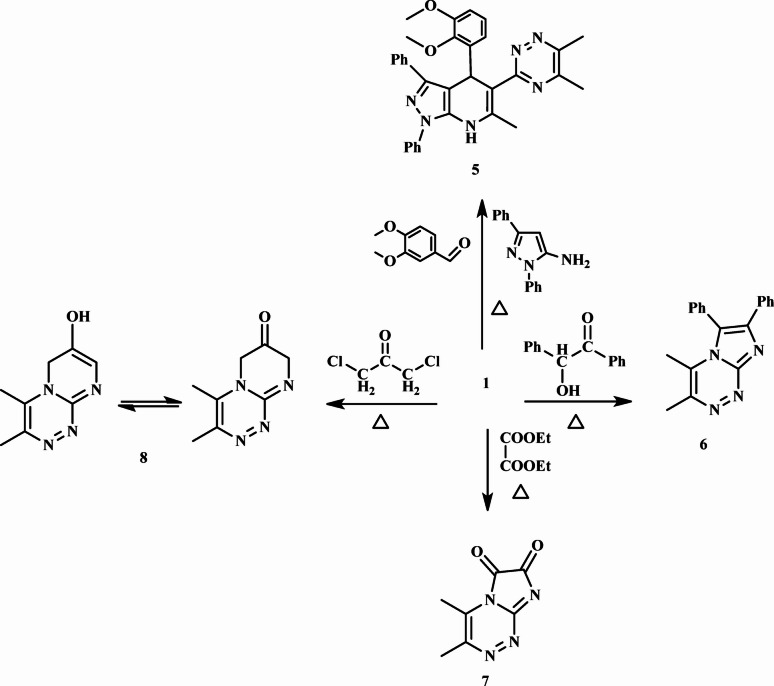
Synthesis of compounds (5–8).

**Fig. 4:**
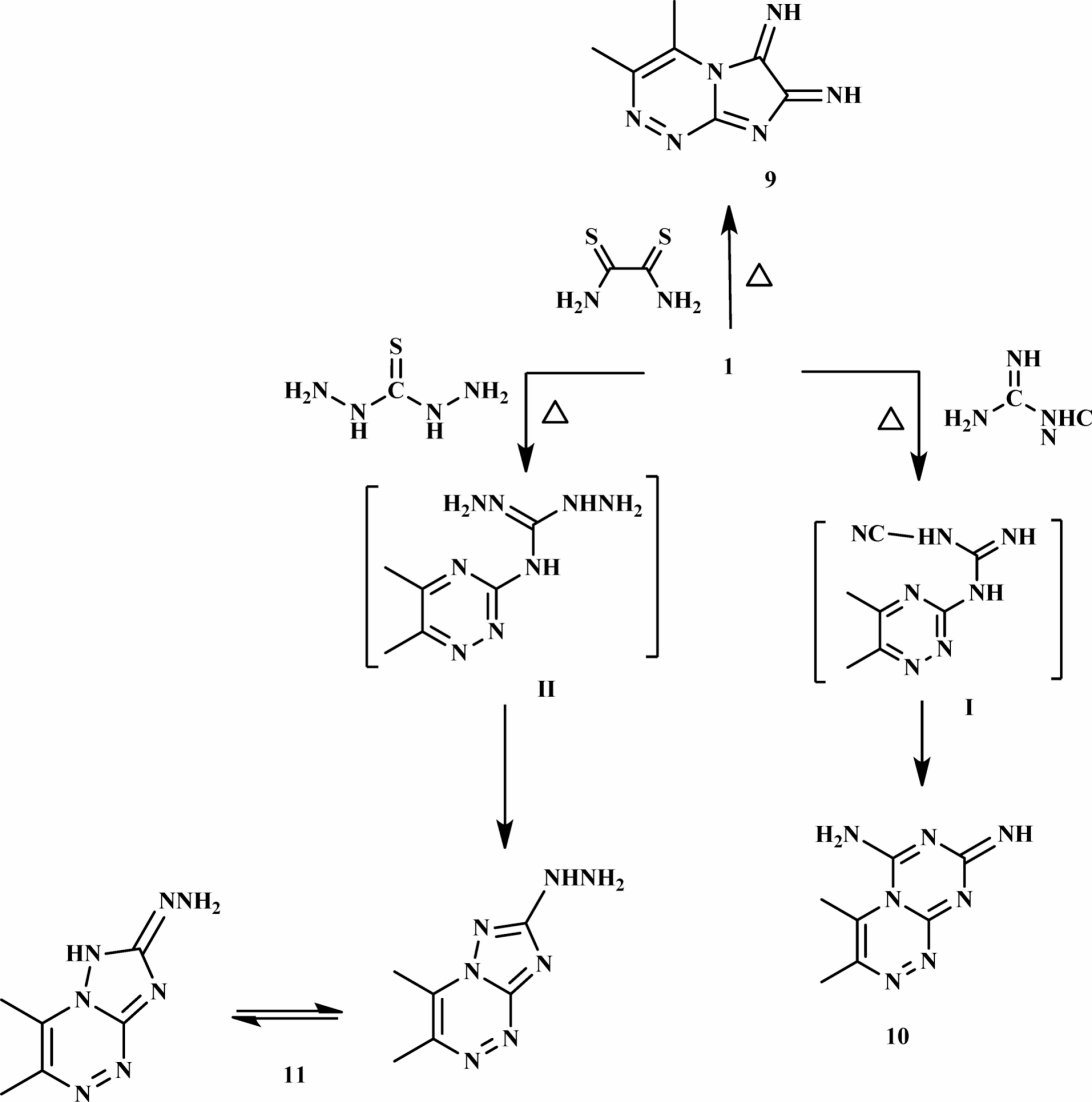
Synthesis of compounds (9–11).

This investigation elucidates the synthesis of novel heterocyclic compounds starting from 5,6-dimethyl-1,2,4-triazin-3-amine (**1**), as illustrated in Figs. 2, 3 and 4. A reaction involving **1** with 4,5,6,7-tetrachlorophthalic anhydride yielded compound **2**, whose structure was verified by elemental analysis (C, H, Cl) and NMR spectroscopy (see Experimental Section). Similarly, treatment of **1** with 2-chloro-*N*-(4-(*N*-(4,6-dimethylpyrimidin-2-yl)sulfamoyl)phenyl)acetamide^38^ produced compound **3**, with the release of HCl. The^1^H NMR spectrum of **3** lacked an NH_2_ signal but displayed signals corresponding to CH_3_, D_2_O-exchangeable NH-triazine, NH-pyrimidine, and NH-phenyl protons.

In a parallel approach, a multicomponent reaction of **1** with 3,4-dimethoxybenzaldehyde and phthalide afforded the fused heterocycles [benzofuro[3’,2’:4,5]pyrimido[2,1-*c*][1,2,4]triazine (**4**), The proposed mechanism involves initial nucleophilic attack by the NH₂ group of compound **1** on the carbonyl group of phthalide, followed by cycloaddition and the elimination of two molecules of H_2_O. The^1^H NMR spectra of **4** displayed characteristic signals: a singlet at *δ* 1.24 ppm for the 2CH_3_-triazine protons, multiplet at *δ* 3.68–3.83 ppm for the 2(OCH_3_) groups, and singlet at *δ* 5.44–5.45 ppm for the CH-pyrimidine protons, alongside signals for aromatic protons (see Experimental Section). The^13^C NMR spectrum of **4** displayed resonances at *δ* 23.2 and 29.5 ppm (2CH_3_-triazine carbons), *δ* 56.1 ppm (CH carbon), *δ* 70.3 ppm (2OCH_3_ carbons), and aromatic carbons (See experimental section) (Fig. 2).

Pyrazolopyridine derivative **5** was synthesized via a multicomponent reaction of 1,5-amino-1,3-diphenylpyrazole, 1,2-propylene oxide, and 3,4-dimethoxybenzaldehyde. The identity and structure of **5** were confirmed by detailed analysis of its ¹H and ¹³C NMR spectra. (refer to the experimental section). Imidazo[2,1-*c*][1,2,4]triazine **6** was obtained through the fusion of **1** and 2-hydroxy-1,2-diphenylethanone. The reaction proceeded via cyclization with the elimination of two molecules of H₂O. The structure of **6** was elucidated using spectroscopic data (see the experimental section). [1,2,4]Triazine-6,7-dione derivative **7** was synthesized by reacting **1** with diethyl oxalate under fusion conditions. The reaction involved cycloaddition followed by the loss of two molecules of ethanol. The structural features of **7** were confirmed by ¹H and ¹³C NMR spectroscopy. The ¹H NMR spectrum exhibited a singlet at *δ* 2.37 ppm, corresponding to the methyl protons on the triazine ring. The ¹³C NMR spectrum showed characteristic signals at *δ* 16.5 and 29.5 ppm (CH₃), *δ* 150.4, 152.5, and 157.4 ppm (aromatic carbons), and *δ* 162.9 and 165.2 ppm for the two carbonyl carbons.

Finally, cycloaddition of **1** to 1,3-dichloropropan-2-one yielded compound **8** accompanied by the liberation of two equivalents of HCl. The ¹H NMR data of **8** exhibited a series of signals: CH₃-triazine protons at *δ* 1.24 ppm (singlet), CH₂-pyrimidinone protons at *δ* 2.51 ppm (multiplet), =CH- proton at *δ* 3.52 ppm (doublet), aromatic/vinyl proton at *δ* 7.21 ppm (singlet), and OH-pyrimidinol proton at *δ* 11.19 ppm (broad singlet) (Fig. 3).

The reaction between **1** and ethane-1,2-bis(thioamide) [dithiooxamide] afforded compound **9** via the elimination of two H₂S molecules. Confirmation of the structure of **9** was achieved by elemental analysis, NMR, and mass spectrometric data, as described in the Experimental section.

Furthermore, the triazine diimine derivative **10** was obtained by reacting compound **1** with cyanoguanidine. This transformation proceeded via the elimination of an ammonia molecule to yield intermediate **I**, followed by cyclization into the final heterocycle **10**. The ¹H and ¹³C NMR analyses of **10** revealed signals indicative of amino (-NH₂), imino (= NH), C = N, and C = NH protons and carbons.

Finally, the exocyclic amino group of compound **1** attacks the electrophilic carbon-sulfur double bond of thiocarbohydrazide, forming intermediate **II**. Subsequently, the endocyclic nitrogen atom in **II** acts as a nucleophile, undergoing rapid intramolecular reaction with the NNH₂ fragment to generate the final product **11**. The ¹H NMR analysis of **11** exhibited the following signals: A singlet at *δ* 1.25 ppm for the CH₃-triazine protons, a singlet at *δ* 5.30 ppm for the NH₂ protons, a singlet at *δ* 6.04 ppm for the NH-triazole proton, and a singlet at *δ* 6.96 ppm for the NH-tautomer proton. (Fig. 4).

### Biological studies

#### Antimicrobial activity

The newly synthesized compounds were evaluated for their antimicrobial activity using the agar well diffusion method against six microorganisms: two Gram-positive bacteria (*Bacillus cereus* MTCC-2296 and *Staphylococcus aureus* MTCC-0459), two Gram-negative bacteria (*Escherichia coli* ATCC-25955 and *Enterobacter cloacae* ATCC-23355), and two fungal strains (*Saccharomyces cerevisiae* ATCC-9763 and *Candida albicans* ATCC-10231). Ciprofloxacin was used as a reference drug against Gram-negative bacteria, Penicillin G for Gram-positive bacteria, and Ketoconazole as the standard antifungal agent. Additionally, the minimum inhibitory concentration (MIC), minimum bactericidal concentration (MBC), and minimum fungicidal concentration (MFC) were determined using the broth dilution method. Preliminary antimicrobial testing results (Table 1) revealed that imidazo derivative **3** exhibited moderate antibacterial effects against *E. coli*, showing inhibition zones (IZs) of 20 mm. Moderate antibacterial effects were observed for compound **4** against *S. aureus* and *E. coli*, with inhibition zones measuring 16 mm and 19 mm, respectively. Compounds **5** and **9** displayed broader-spectrum activity, ranging from moderate to high, against all tested microorganisms.

**Table 1:** In vitro preliminary antimicrobial activities of the new compounds against pathogenic bacteria and fungi.

Compound number	Gram (+) bacteria		Gram (-) bacteria		Fungi	
	Bacillus cereus (MTCC-2296)	Staphylococcus aureus (MTCC-0459)	Escherichia coli (ATCC-25955)	Enterobacter cloacae (ATCC-23355)	Saccharomyces cerevisiae (ATCC-9763)	Candida albicans (ATCC-10231)
**2**	7	N A	12	10	9	11
**3**	14	16	20	14	10	7
**4**	12	16	19	12	15	10
**5**	19	17	22	20	16	18
**6**	12	10	18	14	14	7
**7**	11	8	17	15	10	7
**8**	9	7	14	10	7	12
**9**	19	21	23	17	19	16
**10**	8	6	19	17	N A	6
**Penicillin G**	23	22	–	–	–	–
**Ciprofloxacin**	–	–	30	27	–	–
**Ketoconazole**	–	–	–	–	24	26

NA—not applicable.

### MIC and MBC

The minimum inhibitory concentration (MIC) and minimum bactericidal concentration (MBC) results are summarized in Table 2. Among the tested compounds, compound **9** exhibited the most potent activity against *B. cereus*, with the MIC value of 3.91 µg/mL, followed by compounds **5 and 7**,** which** demonstrated MIC values of 7.81, and 15.6 µg/mL, correspondingly. Compounds **5** and **9** exhibited high MICs against *S. aureus* (15.6 and 3.91 µg/mL, in that order). For *E. coli*, derivatives **4**,** 5**, and **9** showed high MIC values of 3.91, 1.95, and 1.95 µg/mL, while compounds **3**,** 6**, and **10** exhibited moderate MIC values of 15.6 µg/mL. With respect to *E. cloacae*, most compounds showed moderate MICs, except compounds **5**,** 7**, and **10**, which displayed high MICs ranging from 3.91 to 15.6 µg/mL. Regarding the minimum bactericidal concentrations (MBC), all derivatives showed moderate MBC values against the two Gram-positive bacteria, except compound **9**, which had an MBC of 15.6 µg/mL for *B. cereus.* For Gram-negative bacteria, compound **5** showed an MBC value of 15.6 µg/mL against both *E. coli* and *E. cloacae.*

### MIC and MFC

The results for the minimum inhibitory concentrations (MIC) and the minimum fungicidal concentrations (MFC) are illustrated in Table 3. All compounds showed moderate MIC values for both *S. cerevisiae* and *C. albicans*, except compound **9**, which showed the highest MIC of 7.81 µg/mL against *S. cerevisiae*, and compound 5, which had the highest MIC of 7.81 µg/mL against C. *albicans*. On the other hand, all compounds exhibited moderate MFC values for the two fungi except compound 5, which had the highest MFC of 31.3 µg/mL against *C. albicans.*

**Table 2:** In vitro MIC and MBC for the synthesized compounds.

Compound number	MIC (µg/mL)				MBC (µg/mL)			
	Bacillus cereus (MTCC-2296)	Staphylococcus aureus (MTCC-0459)	Escherichia coli (ATCC-25955)	Enterobacter cloacae (ATCC-23355)	Bacillus cereus (MTCC-2296)	Staphylococcus aureus (MTCC-0459)	Escherichia coli (ATCC-25955)	Enterobacter cloacae (ATCC-23355)
**2**	62.5	500	62.5	31.3	125	1500	125	125
**3**	31.3	31.3	15.6	62.5	125	250	31.3	250
**4**	31.3	31.3	3.91	62.5	62.5	62.5	31.3	250
**5**	7.81	15.6	1.95	3.91	31.3	62.5	15.6	15.6
**6**	62.5	62.5	15.6	62.5	250	250	62.5	125
**7**	15.6	125	31.3	15.6	62.5	500	125	62.5
**8**	62.5	125	31.3	62.5	250	500	250	125
**9**	3.91	3.91	1.95	31.3	15.6	31.3	31.3	62.5
**10**	125	125	15.6	7.81	250	250	31.3	31.3

**Table 3:** In vitro MIC and MFC of the newly synthesized compounds.

Compound number	MIC (µg/mL)		MFC (µg/mL)	
	Saccharomyces cerevisiae (ATCC-9763)	Candida albicans (ATCC-10231)	Saccharomyces cerevisiae (ATCC-9763)	Candida albicans (ATCC-10231)
**2**	62.5	31.3	250	125
**3**	31.3	31.3	125	125
**4**	31.3	62.5	125	125
**5**	15.6	7.81	62.5	31.3
**6**	31.3	125	125	500
**7**	125	31.3	500	125
**8**	62.5	31.3	250	125
**9**	7.81	31.3	62.5	62.5
**10**	125	62.5	500	125

### Computational studies

The computations and visualizations for density-functional theory (DFT) were performed applying Gaussian 09 W and GaussView06 ^39^. The B3LYP functional and the 6-31G (d, p) basis set were used to perform all calculations in this study^40^. MOE 2015 was used to identify binding residues, bond lengths, binding energies, and other limitations relevant to compound-protein docking interactions^41^.

### Frontier molecular orbitals (FMOs)

**Fig. 5:**
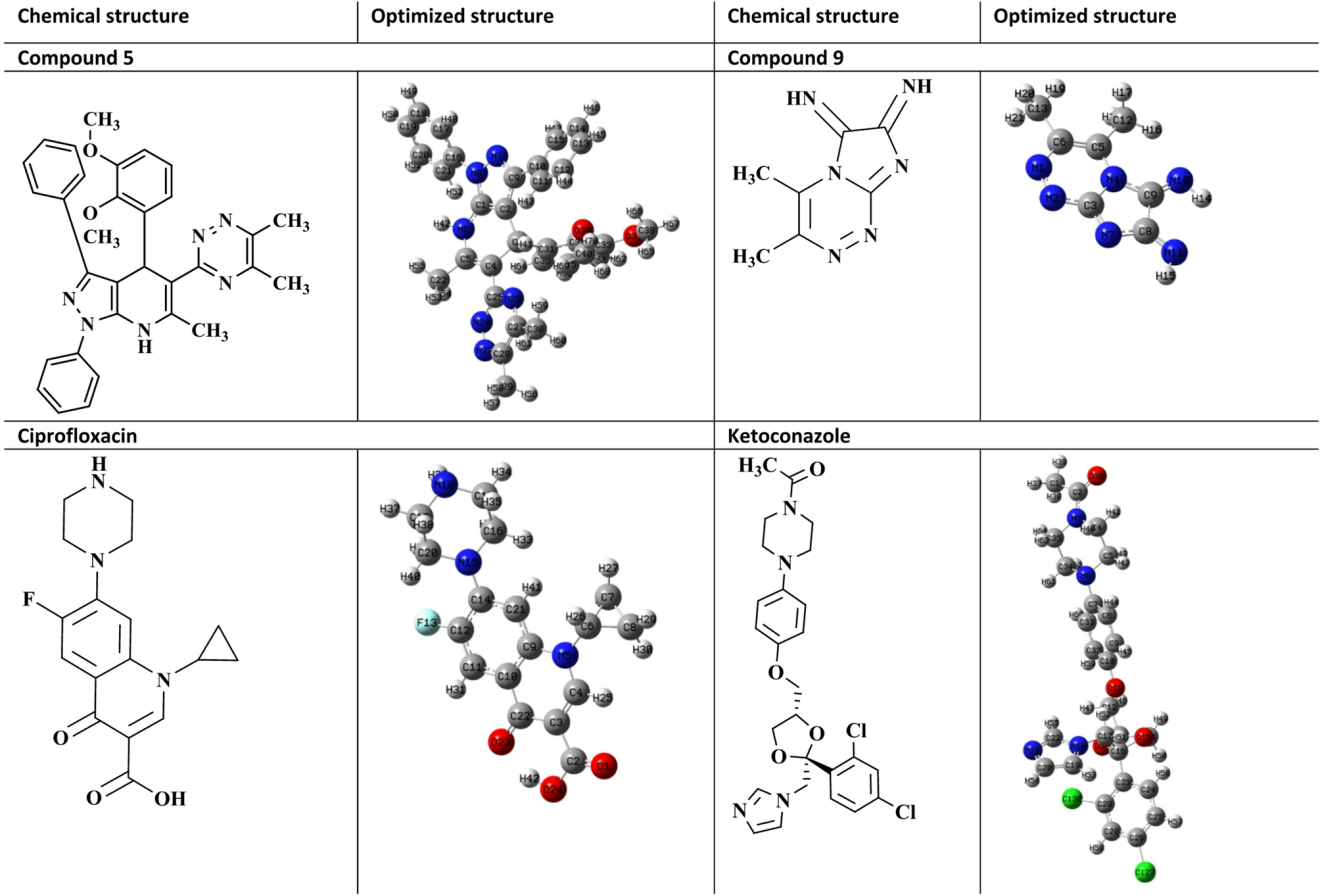
Chemical and optimized structure of compounds 5, 9, ciprofloxacin and ketoconazole. Optimized with DFT-B3LYP/6-31G (d, p).

Chemical reactivity descriptors rely significantly on frontier molecular orbitals (FMOs) to govern molecular electronic properties^42,43^. The highest occupied molecular orbital (HOMO) reflects a molecule’s electron-donating capacity, whereas the lowest unoccupied molecular orbital (LUMO) indicates its electron-accepting capability. Schemes 1–3 show the chemical and optimized compound structures, as well as a graphic depiction of FMOs (Fig. 5).

For compounds **5**, **9**, ciprofloxacin, and ketoconazole, the energy values are (-0.1922, -0.2315, -0.2139, and − 0.1994) eV for E_*HOMO*_, (-0.0566, -0.1196, -0.0609, and − 0.0328) eV for E_*LUMO*_, and (0.1356, 0.1119, 0.153, and 0.1666) eV for ∆E, respectively. Using the HOMO and LUMO orbital energies, the chemical potential (*χ*), electrophilic index (*ω*), chemical hardness (ɳ), and chemical softness (*S*) were calculated.

**Table 4:** Evaluated quantum chemical parameters of compounds **5**, **9**, ciprofloxacin, and ketoconazole.

Molecular descriptors	Dipole moment, µ (Debye)	E_HOMO_ (eV)	E_LUMO_ (eV)	(H–L) ∆E gaps (eV)	X (eV)	ɳ (eV)	S (eV^− 1^)	ω (eV)
**5**	4.3794	− 0.1922	− 0.0566	0.1356	0.1244	0.0678	14.749	0.1141
**9**	4.8861	− 0.2315	− 0.1196	0.1119	0.1755	0.0559	17.873	0.2752
**Ciprofloxacin**	10.516	− 0.2139	− 0.0609	0.153	0.1374	0.0765	13.071	0.1233
**Ketoconazole**	4.2582	− 0.1994	− 0.0328	0.1666	0.1161	0.0833	12.004	0.0809

HOMO, highest occupied molecular orbital; LUMO, lowest unoccupied molecular orbital. *X* = Electronegativity, *η* = Chemical hardness, *S* = Chemical softness, *ω* = Electrophilic index.

Table 4 contains the reactivity descriptions. Compounds **5**, **9**, ciprofloxacin, and ketoconazole have values for their electronic chemical potential (χ = 0.1244, 0.1755, 0.1374, and 0.1161 eV), which represents the tendency of electrons to depart from a stable system. Chemical hardness values of compounds **5**, **9**, ciprofloxacin, and ketoconazole are ɳ = (0.0678, 0.0559, 0.0765, and 0.0833) eV, which indicates resistance to shifting electron distribution and reactivity. The energy decrease caused by the greatest electron flow between the acceptor and donor is represented by the electrophilicity index, ω = (0.1141, 0.2752, 0.1233, and 0.0809) eV.

**Fig. 6:**
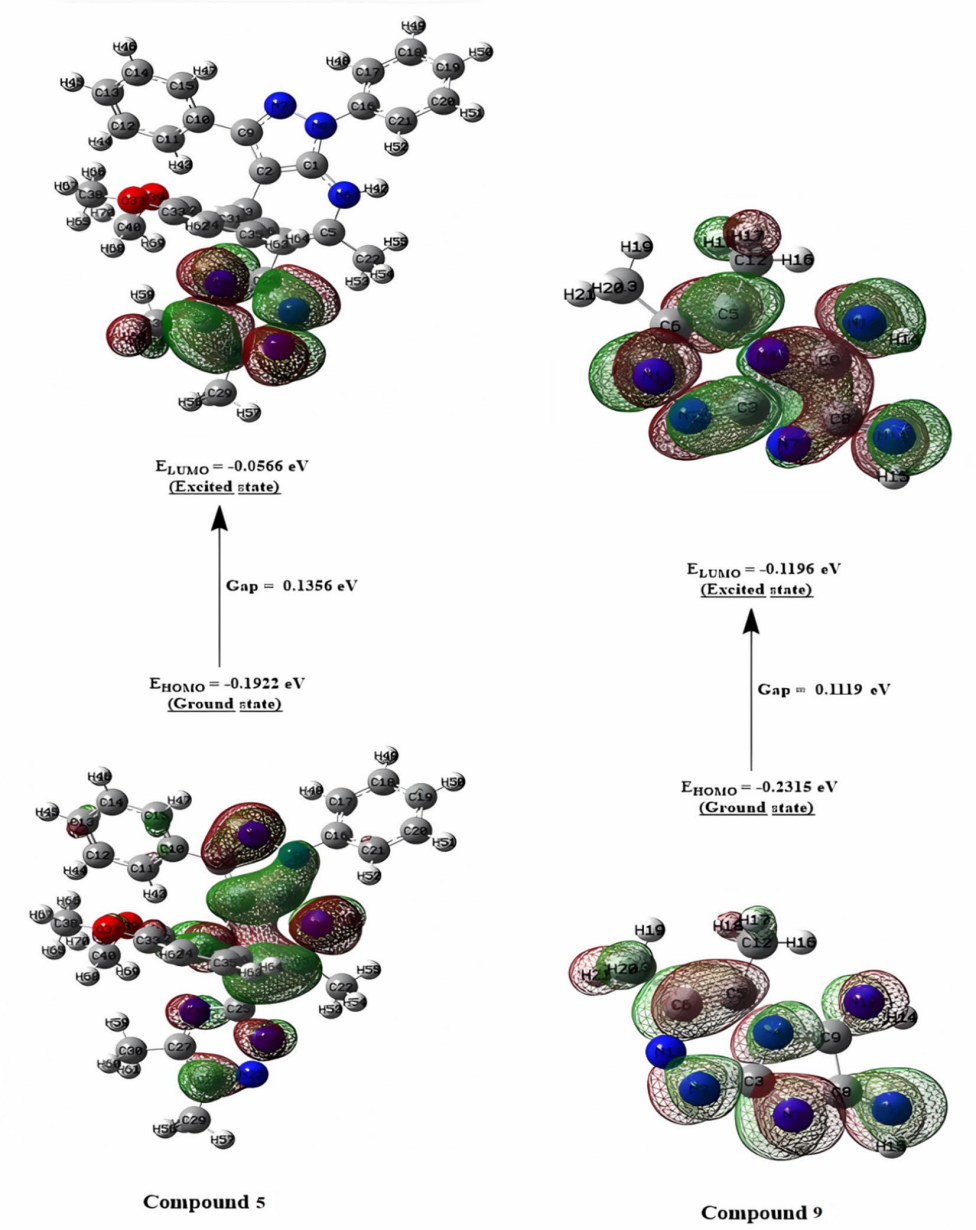
The contour plots of HOMO and LUMO orbitals of compounds **5** and **9**.

**Fig. 7:**
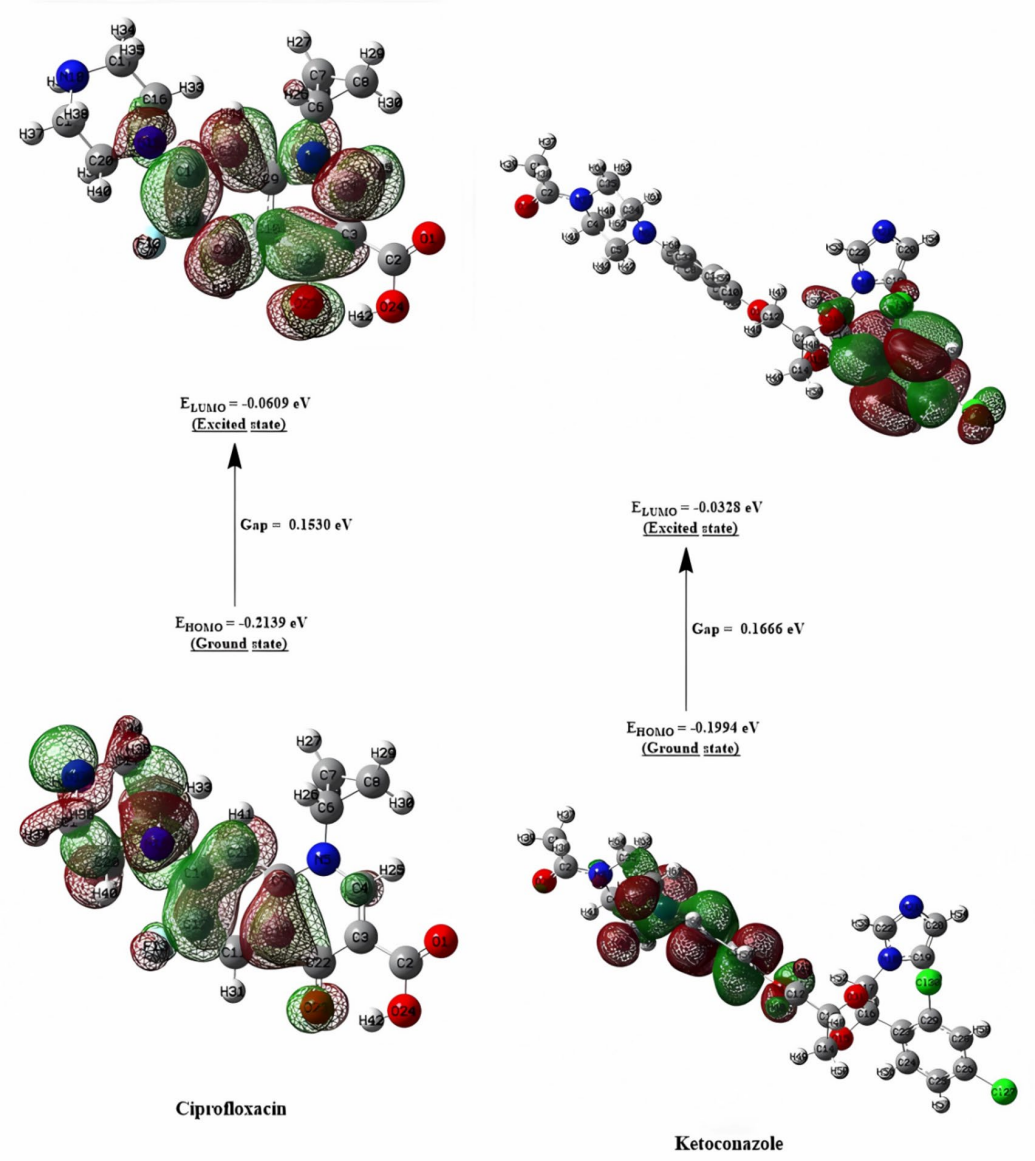
The contour plots of HOMO and LUMO orbitals of ciprofloxacin, and ketoconazole.

The dipole moment values, which are used to assess the polarity of compounds, were obtained, demonstrating that compounds (**5** and **9)** had a high polarity similar to ketoconazole but less than the standard medicine, ciprofloxacin. Because of the low energy difference between their orbitals, the compounds (**5** and **9**) are extremely reactive and unstable. Furthermore, compounds (**5** and **9**) have reduced chemical hardness and higher chemical softness compared to the typical drug. As a result of the computational molecular property discoveries, the studied compounds (**5** and **9**) may exhibit high bio-efficiency due to their strong chemical reactivity (Figs. 6 and 7).

### Molecular Docking studies

Molecular docking simulations were conducted employing MOE 2015 software to investigate the bound residues, bond lengths, binding energies, and other restrictions associated with the interactions between the compounds (**2**–**4**, **6**–**11**) and the target proteins. These simulations were designed to examine compound-protein docking interactions. The target proteins’ crystal structure was retrieved from the protein data bank (http://www.rcsb.org.pdb).

The target protein for the antibacterial docking research was DNA gyrase (PDB ID: 4KTN). The ligands used were penicillin G and ciprofloxacin. The antifungal molecular docking target was the lanosterol 14 alpha-demethylase CYP51 (PDB: 4WMZ). The ligand, ketoconazole, was chosen as the reference medication. Protein preparation was completed with the assistance of MOE 2015. Protein preparation consists of four steps: (a) preprocessing, (b) optimization of hydrogen bonds, (c) removal of water and co-crystallized ligands, and (d) energy minimization. The compounds of triazine derivatives were designed using ChemDraw18.0 software and stored as molfiles (.mol files), which are required for the manufacture of 3D ligands.

In this work, we use molecular docking to investigate the efficacy of the target derivatives (**2**–**4** and **6**–**11**) as inhibitors of DNA gyrase (PDB ID: 4KTN) and CYP51 (PDB: 4WMZ). The docking investigations revealed that compounds (**2–4** and **6–11**) have substantial interactions with 4KTN and 4WMZ. The predicted docking score ranged from − 4.87 to -7.49 kcal/mol for (4KTN) and − 4.74 to -8.73 kcal/mol for (4WMZ) (Table 5 & S1,2), indicating that a more negative docking scores, a more favorable anticipated interaction inside the target proteins’ binding sites. The target proteins’ binding pattern to the tested compounds was expected to be the same, involving the formation of hydrogen bonds, H-pi contacts, and pi-pi stacking interactions with the various amino acids (see the supplementary data).

**Table 5:** Docking interaction data calculations of compounds 5, 9, ciprofloxacin, and ketoconazole inside 4KTN, and 4WMZ active spots.

Compound number	Binding affinity (Kcal/mol)	Affinity Bond strength (Kcal/mol)	Affinity Bond length (in Å from the main residue)	Amino acids	Ligand	Interaction
		4KTN				
5	− 7.49	− 1.1 − 0.8 − 0.6	2.86 3.91 4.04	ARG 78 ILE 80 ILE 80	O 39 6-ring 6-ring	H-acceptor pi-H pi-H
9	− 7.23	− 1.1 − 1.3 − 0.7 − 0.9 − 0.6	3.16 3.31 3.14 3.73 3.98	ASP 75 ASP 75 SER 122 ASN 48 ASN 48	N 18 N 20 N 4 6-ring 5-ring	H-donor H-donor H-acceptor pi-H pi-H
Penicillin G	− 5.57	− 0.8 − 1.0	3.22 2.87	ASN 48 THR 167	C 5 O 20	H-donor H-donor
Ciprofloxacin	− 6.40	− 0.7 − 0.8	3.13 2.65	GLY 119 GLY 79	N 29 O 1	H-donor H-acceptor
		4WMZ				
5	− 8.73	− 0.6 − 0.6 − 1.1	4.51 3.71 4.20	TYR 126 GLY 315 ILE 471	C 26 6-ring 6-ring	H-pi pi-H pi-H
9	− 7.58	− 3.2 − 1.0 − 0.7 − 0.7	2.93 2.92 3.30 4.23	ARG 385 ARG 385 GLY 465 TYR 126	N 4 N 15 N 18 C 11	H-acceptor H-acceptor H-acceptor H-pi
Ketoconazole	− 7.86	− 1.3 − 0.9 − 1.0	4.28 3.85 4.50	CYS 470 CYS 470 CYS 470	C 10 C 15 C 58	H-donor H-donor H-donor

### Docking evaluation against DNA gyrase (PDB ID: 4KTN)

**Fig. 8:**
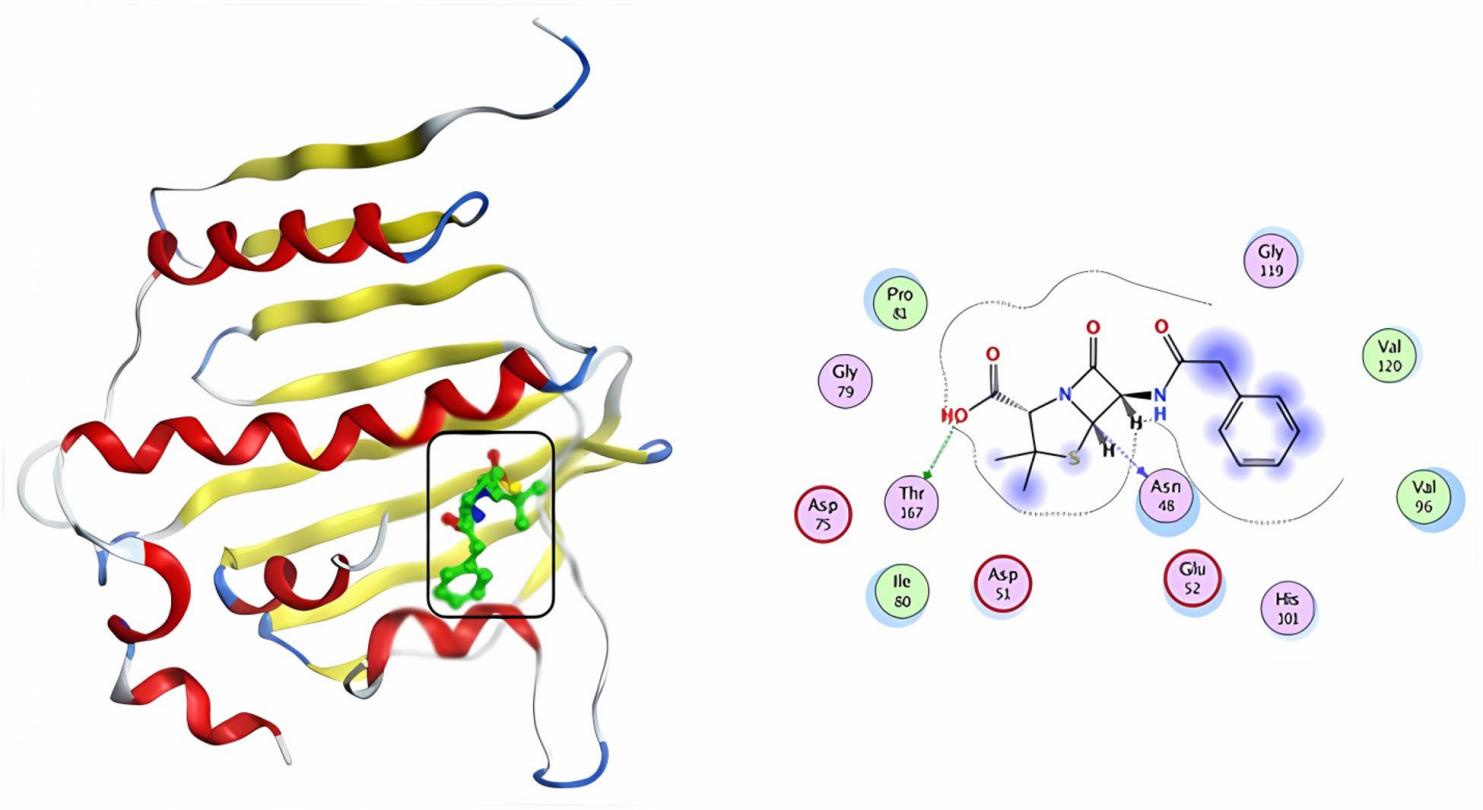
3D & 2D interaction of penicillin G in the active site of 4KTN.

**Fig. 9:**
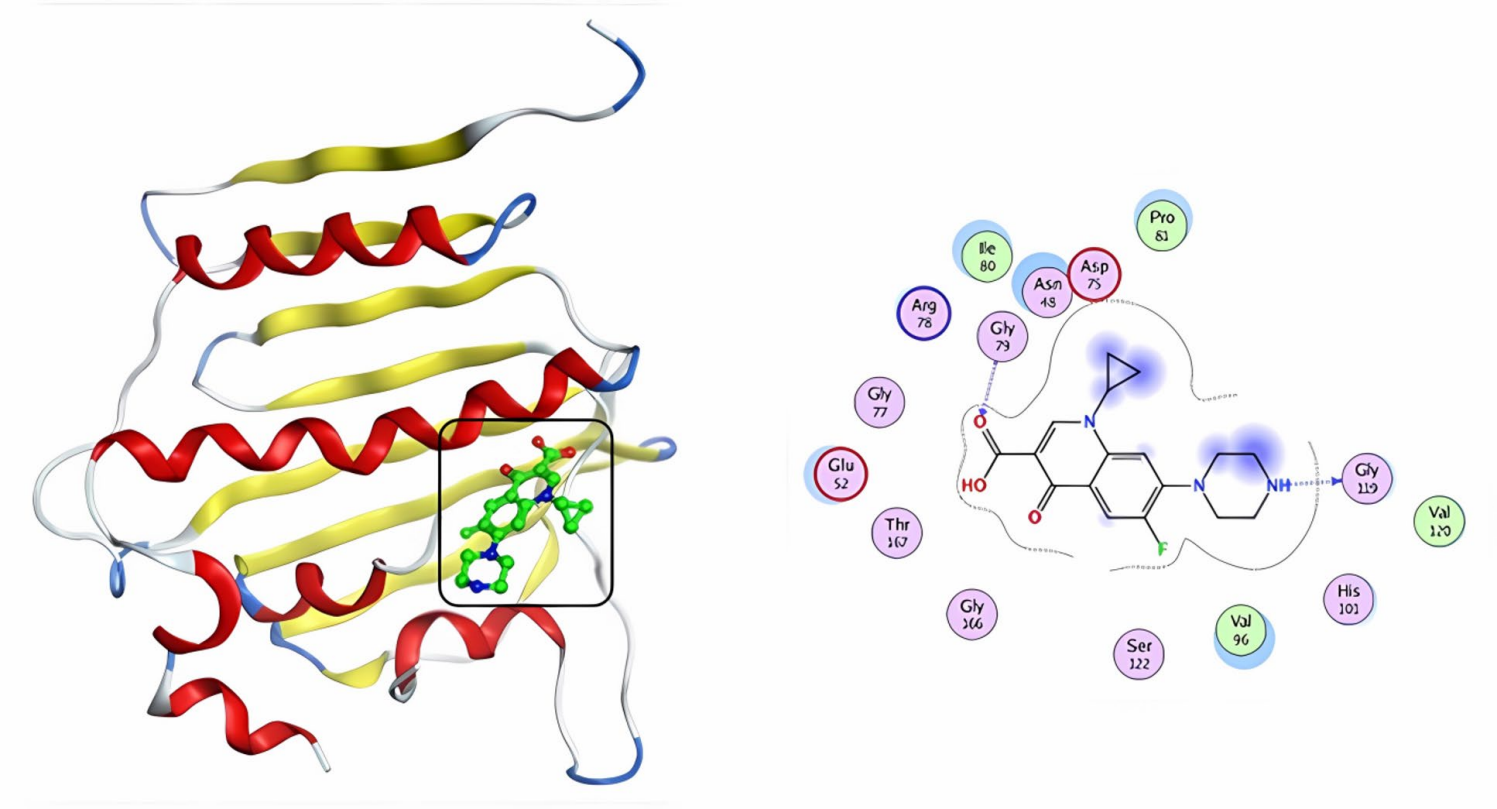
3D & 2D interaction of ciprofloxacin in the active site of 4KTN.

**Fig. 10:**
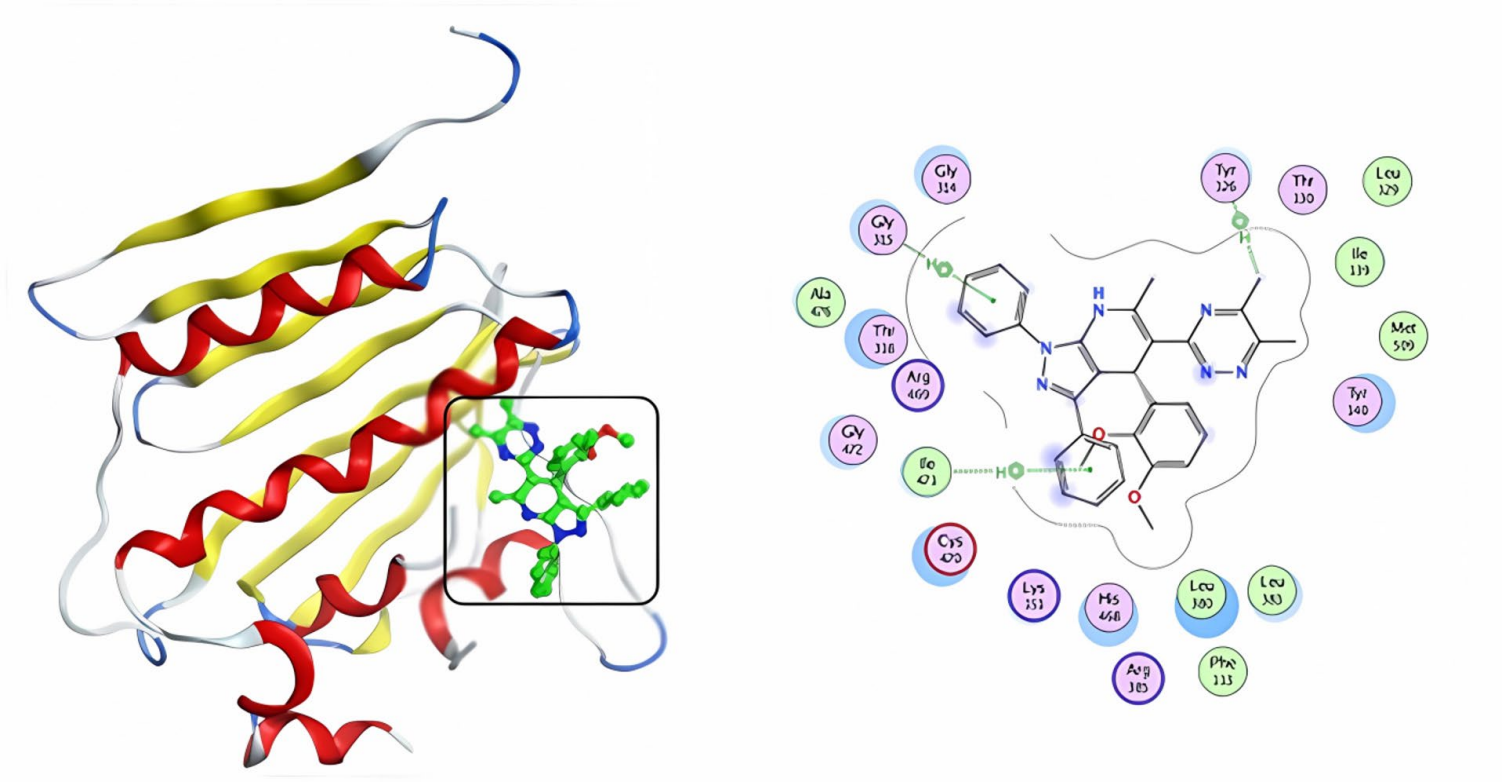
3D & 2D interaction of 5 in the active site of 4KTN.

Table 5 & S1 illustrates the binding energies, bound residues, and bond lengths at the ligand-protein interface. The typical medicine, penicillin G, was re-docked to the pocket’s active site. The docking analysis found that penicillin G has a docking score of -5.57 kcal/mol and two H-bonds with ASN48 and THR167 amino acids (Fig. 8). Ciprofloxacin’s docking score was − 6.40 kcal/mol, and it established two H-bonds with GLY119 and GLY79 residues (Fig. 9). Compound **5** has the highest docking score (S = -7.49 kcal/mol). It also validated binding to the target protein through one hydrogen bond with the ARG78 residue and two pi-H interactions with the ILE80 amino acid (Fig. 10).

**Fig. 11:**
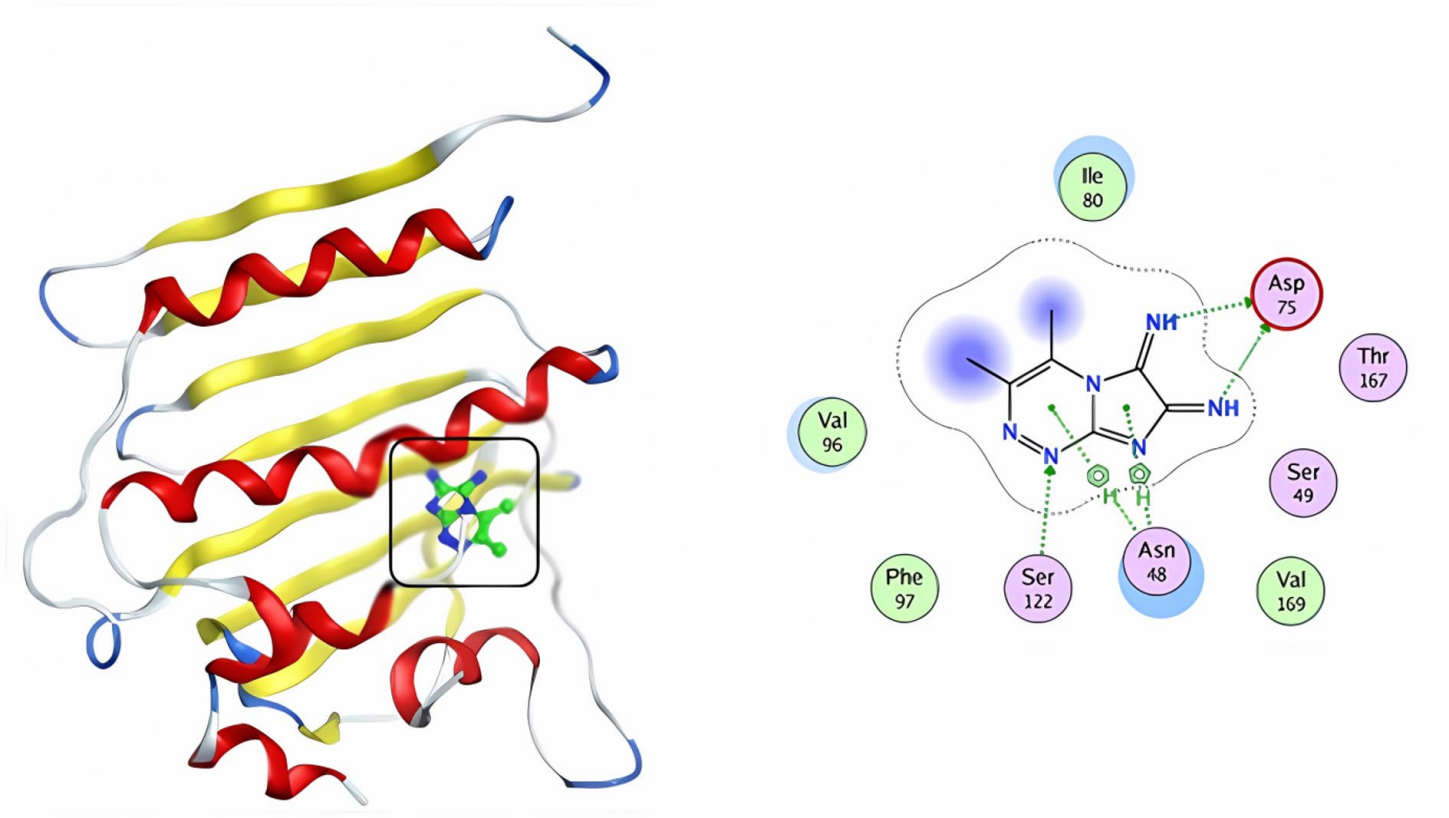
3D & 2D interaction of 9 in the active site of 4KTN.

Finally, compound **9** showed a docking score of S = -7.23 kcal/mol, and the predicted binding pattern identified three hydrogen bonds with amino acid residues including ASP75, and SER122. Further, two pi-H contacts with ASN 48 (Fig. 11). Docking results and the good interactions of the investigated compounds with the 4KTN enzyme indicate that compounds **5** and **9** might be antibacterial inhibitor-targeting therapy options.

### Docking evaluation against the lanosterol 14 alpha-demethylase CYP51 (PDB: 4WMZ)

**Fig. 12:**
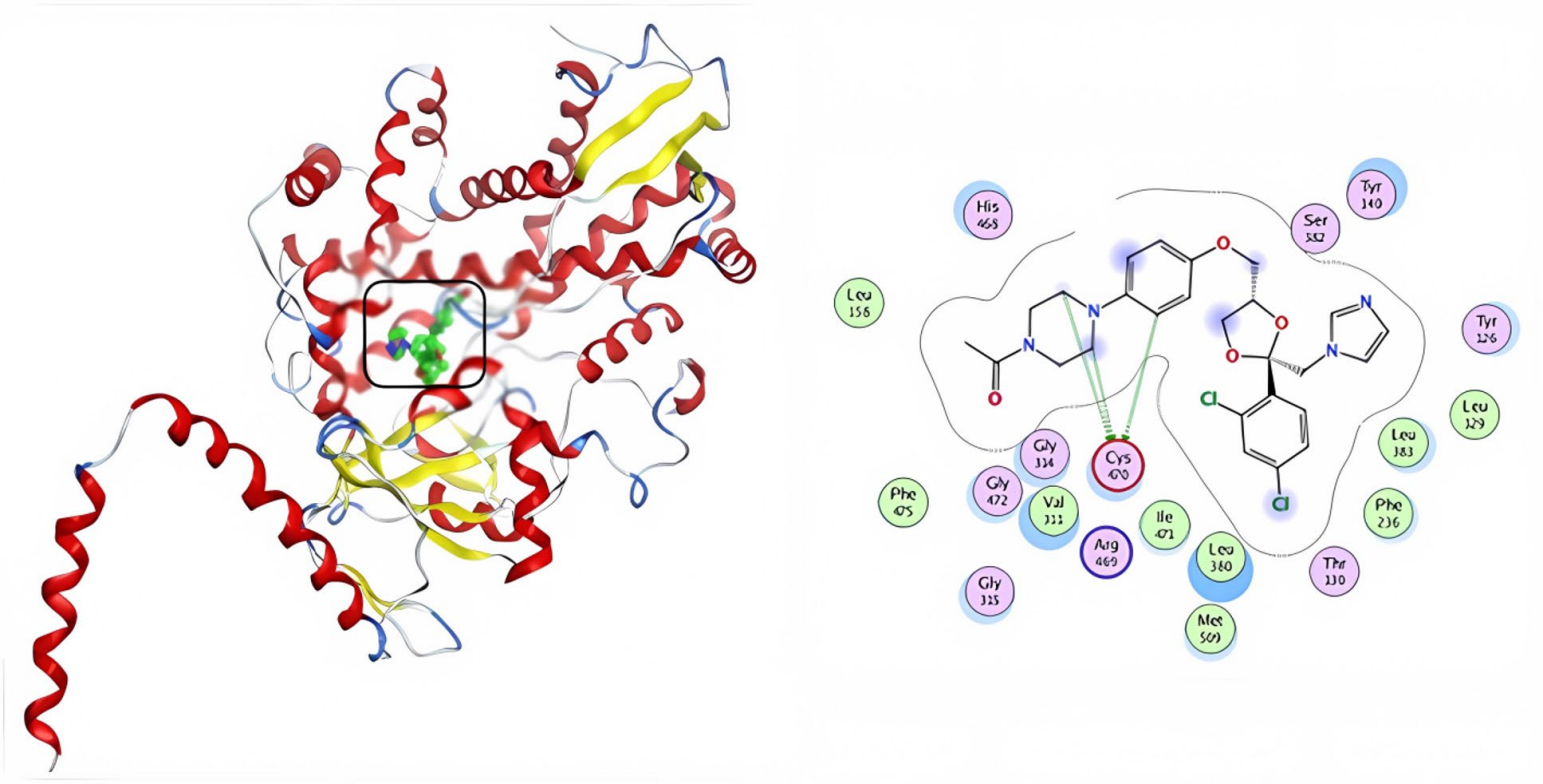
3D & 2D interaction of ketoconazole in the active site of 4WMZ.

**Fig. 13:**
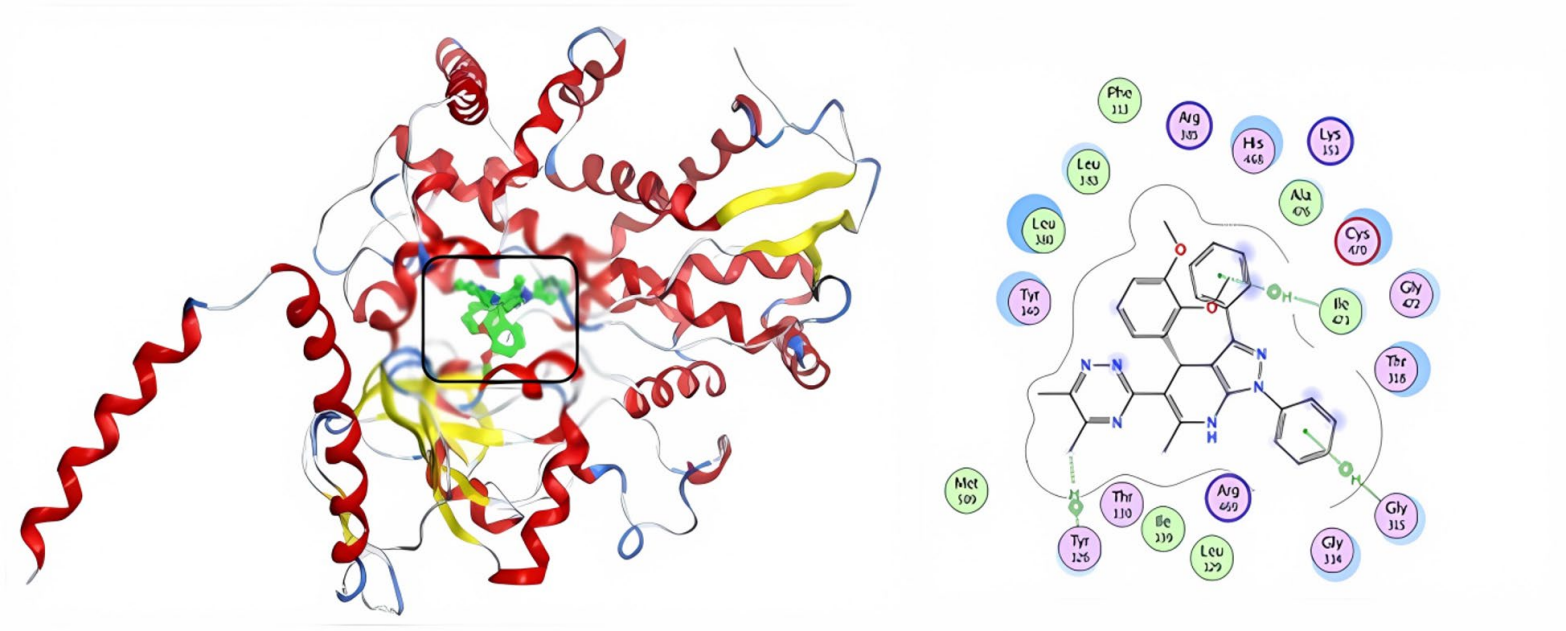
3D & 2D interaction of 5 in the active site of 4WMZ.

**Fig. 14:**
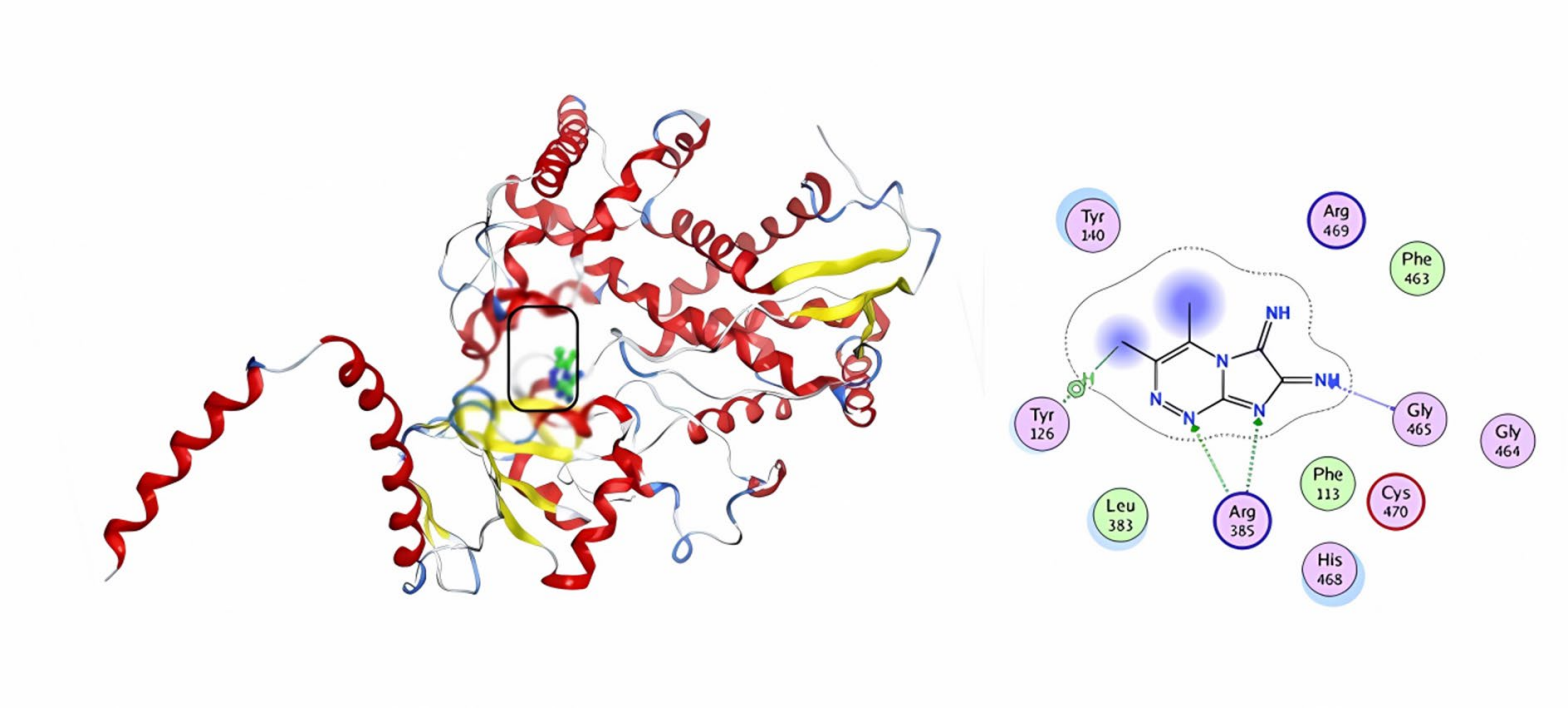
3D& 2D interaction of 9 in the active site of 4WMZ.

Including the active medicinal compound ketoconazole in our docking experiment helped us validate the docking results. Table 5 & S2 show the docking study results in the form of docking scores. Molecular docking experiments revealed that the more powerful compounds (**5** and **9**) in the active site of CYP51 (PDB: 4WMZ) had favorable interactions with amino acids TYR 126, GLY 315, ILE 471, ARG 385, and GLY 465. The docking score of ketoconazole was − 7.86 kcal/mol, and it formed three hydrogen bonds with amino acid CYS 470 (Fig. 12). Compound **5** shows a much lower binding affinity (S = -8.73 kcal/mol) with the target protein than the typical drug ketoconazole, as well as an H-pi interaction with the amino acid TYR126 and two pi-H interactions with the residues GLY315 and ILE471 (Fig. 13). Compound **9** has a docking score (S = -7.58 kcal/mol) similar to ketoconazole; it interacts with amino acids ARG385 and GLY465 via three hydrogen bonds, as well as with TYR126 via H-pi contact (Fig. 14). Compounds **5** and **9** had the highest docking scores, indicating that they may have significant antifungal activity.

### Experimental protocols

#### General information

All melting points were determined in open glass capillaries using an Electrothermal LA 9000 SERIES digital melting point apparatus and are reported without correction. ^1^H and^13^C NMR spectra were recorded at 400 MHz and 100 MHz, respectively, on a Bruker Avance III high-performance digital FT-NMR spectrometer using deuterated dimethyl sulfoxide (DMSO-d_6_) as the solvent. Mass spectra were acquired at 70 eV using a Shimadzu GC/MS-QP-5050 A mass spectrometer at the Regional Center for Mycology and Biotechnology, Al-Azhar University.

### Synthesis of *4*,*5*,*6*,*7-tetrachloro-2-(5*,*6-dimethyl-1*,*2*,*4-triazin-3-yl)isoindoline-1*,*3-dione(2)*

In a 50 ml round-bottom flask, equal amounts of **1**^33^ (1.24 g, 10 mmol) and 4,5,6,7-tetrachlorophthalic anhydride (2.86 g, 10 mmol) were heated until the contents melted; the reaction was maintained at a temperature of 180–188℃ for 3 h. The fused mass was treated with hot ethanol, cooled, and the solid was collected by filtration and recrystallized from ethanol to afford **2**.

Grey solid, 68% yield, m.p: >360 ℃. ^1^H NMR (400 MHz, DMSO-*d*_6_, *δ*, ppm): 1.24 (s, 6 H, 2CH_3_-triazine); ^13^C NMR (100 MHz, DMSO-*d*_6_, *δ*, ppm): 13.61, 29.51 2(CH_3_), 128.48, 129.21, 129.52, 132.44, 135.89, 138.42 (Ar-C), 164.93, 165.24 (2 C = O), 165.61, 165.82, 166.12 (3 C = N). Anal. Calcd for C_13_H_6_Cl_4_N_4_O_2_ (392.02): C, 39.83; H, 1.54; Cl, 36.17; N, 14.29%. Found: C, 39.88; H, 1.58; Cl, 36.20; N, 14.33%.

### Synthesis of *2-((5*,*6-dimethyl-1*,*2*,*4-triazin-3-yl)amino)-N-(4-(N-(4*,*6-dimethylpyrimidin-2-yl)sulfamoyl)phenyl)acetamide* (3)

A mixture of **1**^33^ (1.24 g, 10 mmol) and 2-chloro-*N*-(4-(*N*-(4,6-dimethylpyrimidin-2-yl)sulfamoyl)phenyl)acetamide^38^ (3.54 g, 10 mmol) was heated at 180–187℃ for 14 h. After cooling, the resulting solid was filtered, washed with ethanol, dried, and recrystallized from ethanol to afford compound **3.**

Grey solid, 75% yield, m.p: >360 ℃. ^1^H NMR (400 MHz, DMSO-*d*_6_, *δ*, ppm): 1.24 (s, 12 H, 4CH_3_-triazine and pyrimidine), 2.9 (s, 2 H, CH_2_), 6.04 (s, 1H, NH, D_2_O exchangeable), 7.16–7.36 (m, 5 H, Ar-H), 8.01 (s, 2 H, 2NH, D_2_O exchangeable). Anal. Calcd for C_19_H_22_N_8_O_3_S (442.49); C, 51.57; H, 5.01; N, 25.32; S, 7.25%. Found: C, 51.60; H, 5.05; N, 25.36; S, 7.28%.

### Synthesis of. *6-(3*,*4-Dimethoxyphenyl)-3*,*4-dimethyl-6 H-benzofuro[3’*,*2’:4*,*5]pyrimido[2*,*1-c][1*,*2*,*4]triazine* (4)

An equimolar mixture of **1**^33^ (1.24 g, 10 mmol), 3,4-dimethoxybenzaldehyde (1.66 g, 10 mmol), and phthalide (1.34 g, 10 mmol) was heated until the contents melted. The reaction was maintained at 180–189 ℃ for 6 h. The mixture was treated with ethanol, and cooled, the solid that was obtained was collected by filtration and recrystallized from ethanol to afford compound **4.**

Black powder, 78% yield, m.p: >360 ℃. ^1^H NMR (400 MHz, DMSO-*d*_6_, *δ*, ppm): 1.24 (s, 6 H, 2CH_3_-triazine), 3.81, 3.83 (2s, 6 H, 2OCH_3_), 5.44 (s, 1H, CH-pyrimidine), 7.59–7.88 (m, 7 H, Ar-H); ^13^C NMR (DMSO-*d*_*6*_, *δ*, ppm): ): 29.5 (2CH_3_-triazine), 56.1 (2OCH_3_), 70.3 (CH-pyrimidine), 119.6, 121.1, 123.4, 125.3, 129.4, 132.6, 134.7 (Ar-C). MS (m/z, %): 388.47 (M^+^, 15), 220.81 (100).Anal. Calcd for C_22_H_20_N_4_O_3_ (388.42): C, 68.03; H, 5.19; N, 14.42%. Found: C, 67.95; H, 5.15; N, 14.41%.

### Synthesis **of*4-(2***,***3-dimethoxyphenyl)-5-(5***,***6-dimethyl-1***,***2***,***4-triazin-3-yl)-6-methyl-1***,***3-diphenyl-4***,***7-dihydro-1 H-pyrazolo[3***,***4-b]pyridine*(5)**

An equal molar amount of **1**^33^ (1.24 g, 10 mmol), 3,4-dimethoxybenzaldehyde (1.66 g, 10 mmol), 1,2-propylene oxide (0.58 g, 10 mmol), and 1,3-diphenyl-1*H*-pyrazol-5-amine (2.35 g, 10 mmol) were heated until molten and maintained at a temperature of 170–178 °C for 6 h. The mixture was treated with boiled ethanol, cooled, and the product was filtered and recrystallized from ethanol to obtain compound **5**.

Brown powder, 65% yield, m.p.: > 360 ℃. ^1^H NMR (400 MHz, DMSO-*d*_*6*_, *δ*, ppm): 1.23 (s, 9 H, 3CH_3_), 3.62, 3.65 (2s, 6 H, 2(OCH_3_)), 4.29 (s, 1H, CH-pyridine), 6.11 (s, 1H, NH, D_2_O exchangeable), 7.01–8.05 (m, 13H, Ar-H); ^13^C NMR (DMSO- *d*_*6*_,*δ*,*ppm*):15.3, 20.1, 29.4 (3CH_3_), 55.9 (2(OCH_3_)), 106.5, 110.2, 111.7, 124.3, 131.2, 134.1, 139.4 (Ar-C). Anal. Calcd for C_32_H_30_N_6_O_2_ (530.62): C, 72.43; H, 5.70; N, 15.85%. Found: C, 72.47; H, 5.75; N, 15.90%.

### Synthesis of *3*,*4-dimethyl-6*,*7-diphenylimidazo[2*,*1-c][1*,*2*,*4]triazine* (6)

In a 50 ml round-bottom flask, equal amounts of **1**^33^ (1.24 g, 10 mmol) and 2-hydroxy-1,2-diphenylethanone (2.12 g, 10 mmol) were combined. The mixture was heated until complete melting and then maintained at 160–165 °C for 6–8 h. After completion, the fused mass was treated with boiled ethanol, cooled, and the solid was collected by filtration and recrystallized from ethanol to afford **6.**

Brown powder, 65% yield, m.p: >360 ℃. ^1^H NMR (400 MHz, DMSO-*d*_6_, *δ*, ppm): 1.24 (s, 6*H*, 2CH_3_-triazine), 6.95–7.95 (m, 10 H, Ar-H). MS (m/z, %): 300.08 (M^+^, 27), 257.30 (100). Anal. Calcd for C_19_H_16_N_4_ (300.36): C, 75.98; H, 5.37; N, 18.65%. Found: C, 75.97; H, 5.33; N, 18.66%.

### **General method for the synthesis of compounds 7–8**

These compounds were synthesized by fusion of **1**^33^ (1.24 g, 10 mmol), diethyl oxalate (1.46 g, 10 mmol), or 1,3-dichloropropan-2-one (1.26 g, 10 mmol) until the contents melt. The reaction was maintained at 170–173℃ for 6–8 h. The mixture was treated with ethanol and cooled; the solid that was obtained was collected by filtration and recrystallized from EtOH to afford the final triazine derivatives **7**, and **8**, respectively.

### *3*,*4-Dimethylimidazo[2*,* 1-c][1*,* 2*,* 4]triazine-6*,*7-dione* (7)

Brownish powder, 75% yield, m.p: >360 ℃. ^1^H NMR (400 MHz, DMSO-*d*_6_, *δ*, ppm): 1.25 (s, 6 H, 2CH_3_-triazine); ^13^C NMR (DMSO-*d*_6_, *δ*, ppm): 16.5, 29.5 (2CH_3_-triazine), 162.9, 165.2 (2 C = O). MS (m/z, %): 178.34 (M^+^, 19), 120.16 (100). Anal. Calcd for C_7_H_6_N_4_O_2_ (178.15): C, 47.19; H, 3.39; N, 31.45%. Found: C, 47.10; H, 3.36; N, 31.40%.

### *3*,*4-Dimethyl-6 H-pyrimido[2*,*1-c][1*,*2*,*4]triazin-7(8 H)-one* (8)

Black powder, 80% yield, and m.p: >360 ℃. ^1^H NMR (400 MHz, DMSO-*d*_6_, *δ*, ppm): 1.24 (s, 6 H, 2CH_3_-triazine), 2.51 (s, 2 H, CH_2_- pyrimidinol), 3.52 (s, 2 H, CH_2_-pyrimidinone), 7.21 (s, 1H, =CH-pyrimidinol), 11.19 (br.s, 1H, OH-pyrimidinol, D_2_O exchangeable). MS (m/z, %): 178.89 (M^+^, 51), 116.95 (100).Anal. Calcd for C_8_H_10_N_4_O (178.19): C, 53.92; H, 5.66; N, 31.44%. Found: C, 53.66; H, 5.60; N, 31.30%.

### General method for the synthesis of compounds 9–11

An equimolar mixture of **1**^33^ (1.24 g, 10 mmol) and 10 mmol from each of ethane-1,2-bis(thioamide) [dithiooxamide] (1.20 g) /or, cyanoguanidine (8.4 g) /or thiocarbohydrazide (1.06 g)] was heated until the contents melted; the reaction mixture was maintained at (180–185) ℃ for 6 h. The mixture was treated with boiled ethanol and cooled, and the solid obtained was collected by filtration and recrystallized from the proper solvent.

### *3*,*4-Dimethylimidazo[2*,*1-c][1*,*2*,*4]triazine-6*,*7-diimine* (9)

Black powder, 68% yield, and m.p: >360 ℃. ^1^H NMR (400 MHz, DMSO-*d*_6_, *δ*, ppm): 1.25 (s, 6 H, 2CH_3_-triazine), 6.99 (s, 1H, =NH, D_2_O exchangeable), 7.03 (s, 1H, =NH, D_2_O exchangeable). MS (m/z, %): 176.24 (M^+^, 31), 84.35 (100).Anal. Calcd for C_7_H_8_N_6_ (176.18): C, 47.72; H, 4.58; N, 47.70%. Found: C, 47.66; H, 4.53; N, 47.66%.

### *8-Imino-3*,*4-dimethyl-8 H-[1*,*3*,*5]triazino[2*,*1-c][1*,*2*,*4]triazin-6-amine* (10)

Gray powder, 78% yield, and m.p: >360 ℃. ^1^H NMR (400 MHz, DMSO-*d*_6_, *δ*, ppm): 1.24 (s, 6 H, 2CH_3_-triazine), 6.04 (s, 2 H, NH_2_, D_2_O exchangeable), 7.10 (s, 1H, =NH, D_2_O exchangeable); ^13^C NMR (DMSO-*d*_6_, *δ*, ppm): 158.39 (C = NH), 167.71 (C = N). MS (m/z, %): 191.02 (M^+^, 20), 185.09 (100). Anal. Calcd for C_7_H_9_N_7_ (191.19): C, 43.97; H, 4.74; N, 51.28%. Found: C, 43.97; H, 4.71; N, 51.30%.

### *7-Hydrazinyl-3*,*4-dimethyl-[1*,*2*,*4]triazolo[5*,*1-c][1*,*2*,*4]triazine* (11)

Black powder, 68% yield, and m.p: >360 ℃. ^1^H NMR (400 MHz, DMSO-*d*_6_, *δ*, ppm): 1.25 (s, 6 H, 2CH_3_-triazine), 5.30 (s, 2 H, NH_2_, D_2_O exchangeable), 6.04 (s, 1H, NH- triazolo, D_2_O exchangeable), 6.96 (s, 1H, NH-tautomer, D_2_O exchangeable). MS (m/z, %): 179.08 (M^+^, 21), 141.05 (100). Anal. Calcd for C_6_H_9_N_7_ (179.18): C, 40.22; H, 5.06; N, 54.72%. Found: C, 40.20; H, 5.02; N, 54.72%.

### Biological studies

#### Antimicrobial activity

The antibacterial and antifungal activities were assessed through the agar disk diffusion method against four bacterial and two fungal strains^44^. Standard antibiotics, including penicillin G, ciprofloxacin (for Gram-positive and Gram-negative bacteria), and ketoconazole (as an antifungal control), were all utilized.

#### MIC measurement

The minimum inhibitory concentration (MIC) and minimum bactericidal concentration (MBC) were evaluated via the microdilution assay (broth dilution)^45^. In a microdilution plate, there is one quality control (QC) antibiotic and two-fold stepwise dilutions of the evaluated compounds (up to ten). Starting at a concentration of 1000 µg, then 500 µg, 250 µg, 125 µg, 62.5 µg, 31.3 µg, 7.81 µg, 3.91 µg, etc., until you reach 1.95 µg. The inoculum is made by removing a small number of colonies from an agar plate utilizing a sterile brush. An overnight broth culture was prepared, from which a 0.5 McFarland standard was made and dilutedinto medium (at 580 nm, with an optical density of 0.1). Before leaving the microdilution plate to incubate overnight, add the inoculum and the test compounds that have been serially diluted. Find out the MIC value by analyzing the microdilution assay plate. Identify the MBC and MFC by plating a section of each well onto the appropriate agar media, followed by incubation and subsequent inspection for colony formation. The MIC was determined to be the concentration of the substance at which no discernible growth was seen following 48 h of inoculation. The MBC showed the lowest concentration at which no discernible growth was seen following 96 h of inoculation.

## Conclusions

In this study, a novel class of heterocyclic compounds containing a triazine moiety was synthesized by reacting 5,6-dimethyl-1,2,4-triazine (**1**) with different reagents. The chemical structures of the obtained derivatives were confirmed using elemental analysis, infrared (IR) spectroscopy, nuclear magnetic resonance (NMR), and mass spectrometry (MS). Antibacterial and antifungal activities of the newly synthesized compounds were evaluated in vitro against a panel of microorganisms, including two Gram-positive bacteria, two Gram-negative bacteria, and two fungal strains. Among the derivatives, pyrazolopyridine **5** and pyrazolotriazine **9** exhibited the most potent activity, with MIC values ranging from 1.95 to 31.3 µg/mL against both Gram-positive and Gram-negative bacteria. Remarkably, compound **9** demonstrated the most potent antifungal activity against Saccharomyces cerevisiae, with an MIC of 7.81 µg/mL. Compounds **5** and **9** were further optimized using density functional theory (DFT) at the B3LYP/6-31G(d, p) level. All calculations were carried out with Gaussian 09 W and visualized using Gauss View 6.0. Frontier molecular orbital (FMO) analysis revealed intramolecular charge transfer (ICT) in both compounds, with band gap energies (ΔE) of 0.1356 eV (compound **5**) and 0.1119 eV (compound **9**), indicating enhanced electronic properties. Finally, molecular docking studies against target microbial proteins (PDB IDs: 4KTN and 4WMZ) revealed strong binding interactions for compounds **5** and **9**, as evidenced by their exceptionally low binding energies. These results suggest their potential as antimicrobial agents targeting key microbial enzymes.

## Supplementary Information

Below is the link to the electronic supplementary material.

Supplementary Material 1

## Data Availability

All data generated or analyzed during this study are included in this manuscript and its supplementary information file.
