# Supplementary material for: Synthesis, antimicrobial evaluation, and computational investigation of new triazine-based compounds via DFT and molecular docking
Source: Sci Rep. 2025 Nov 29;15:42921. doi: 10.1038/s41598-025-27847-4 (PMC12673065; doi:10.1038/s41598-025-27847-4)
Supplement: Supplementary file 1 — Supplementary Material 1 [file 41598_2025_27847_MOESM1_ESM.docx]

**Synthesis, Antimicrobial Evaluation, and Computational Investigation of New Triazine-Based Compounds via DFT and Molecular Docking**

**Aisha O. Hussain | Aisha Y. Hassan** **| Anhar Abdel‐Aziem* | Eman S. Abou-Amra**

Department of Chemistry, Faculty of Science (Girl's Branch), Al‐Azhar University, P.O. Box 11754, Yousef Abbas Street, Nasr City, Cairo, Egypt.

***Correspondence: Anhar Abdel-Aziem, email: [anhar@azhar.edu.eg](mailto:anhar@azhar.edu.eg)**


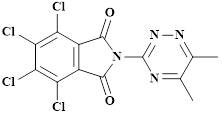

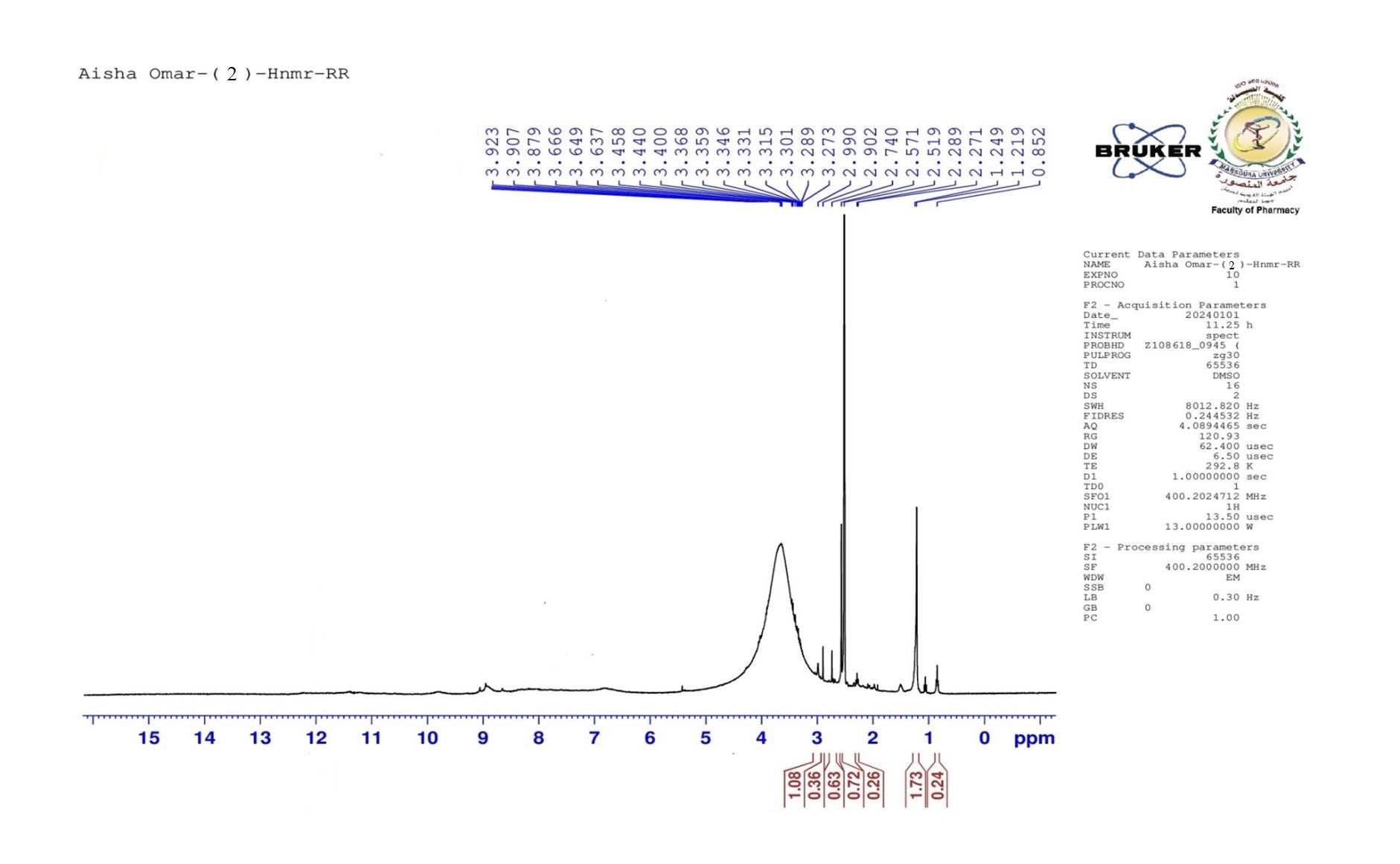


**Fig(S1)**: ^1^H NMR for compound **2**.


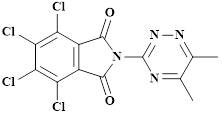

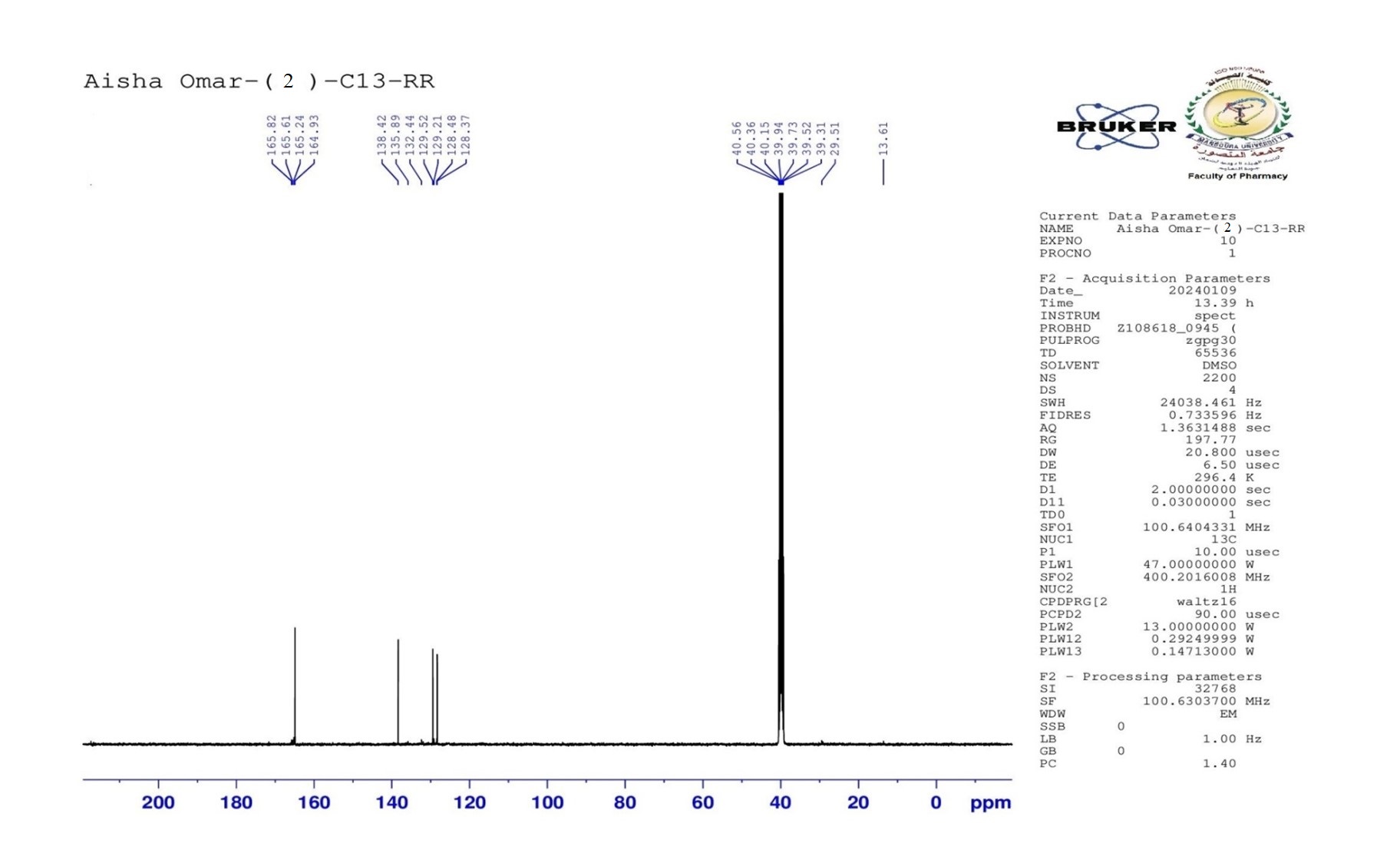
.

**Fig(S2)**: ^13^C NMR for compound **2**

**
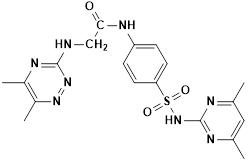

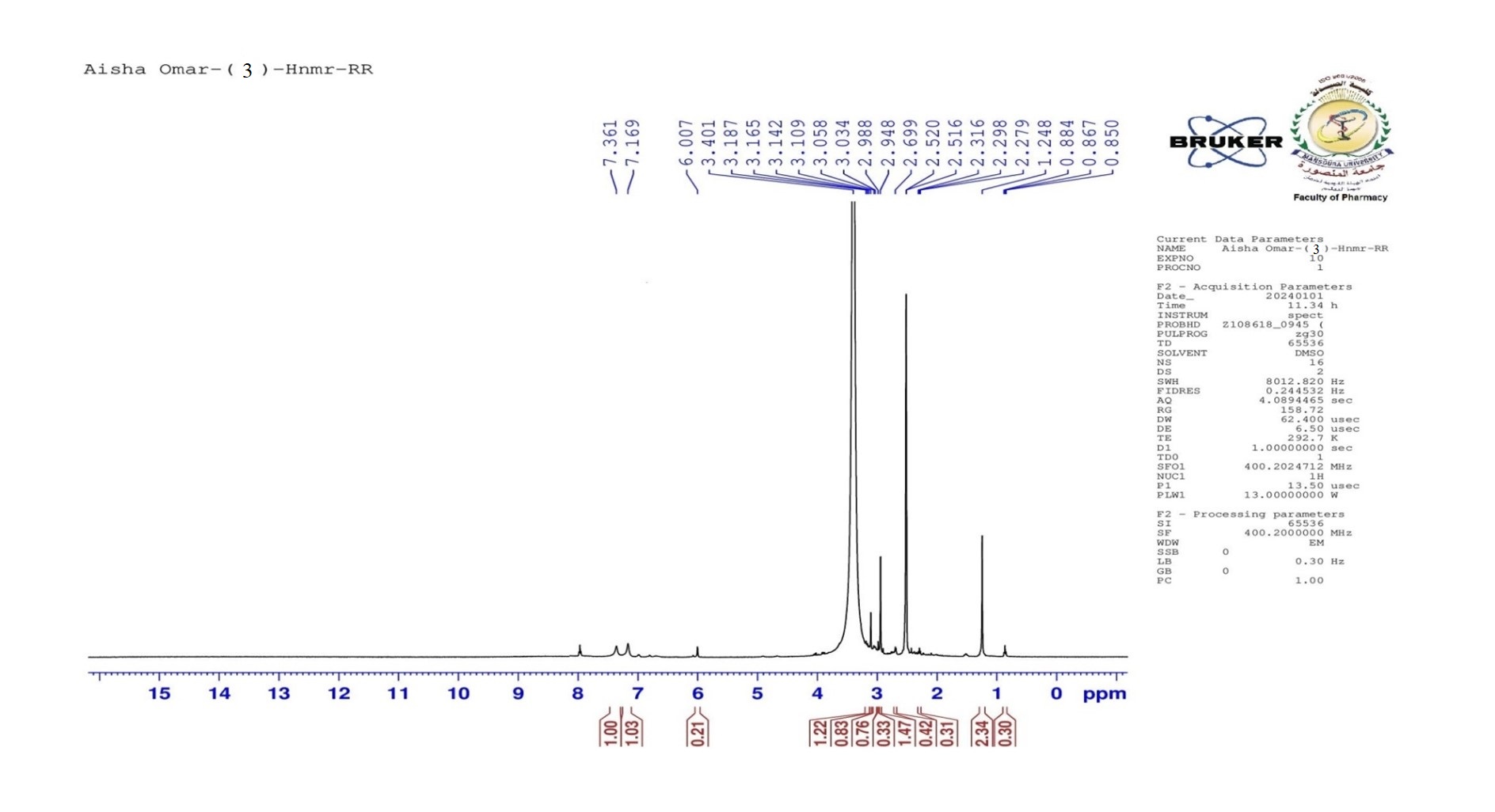
**

**Fig(S3)**: ^1^H NMR for compound **3**.


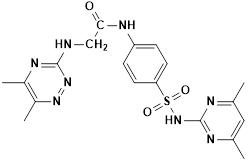

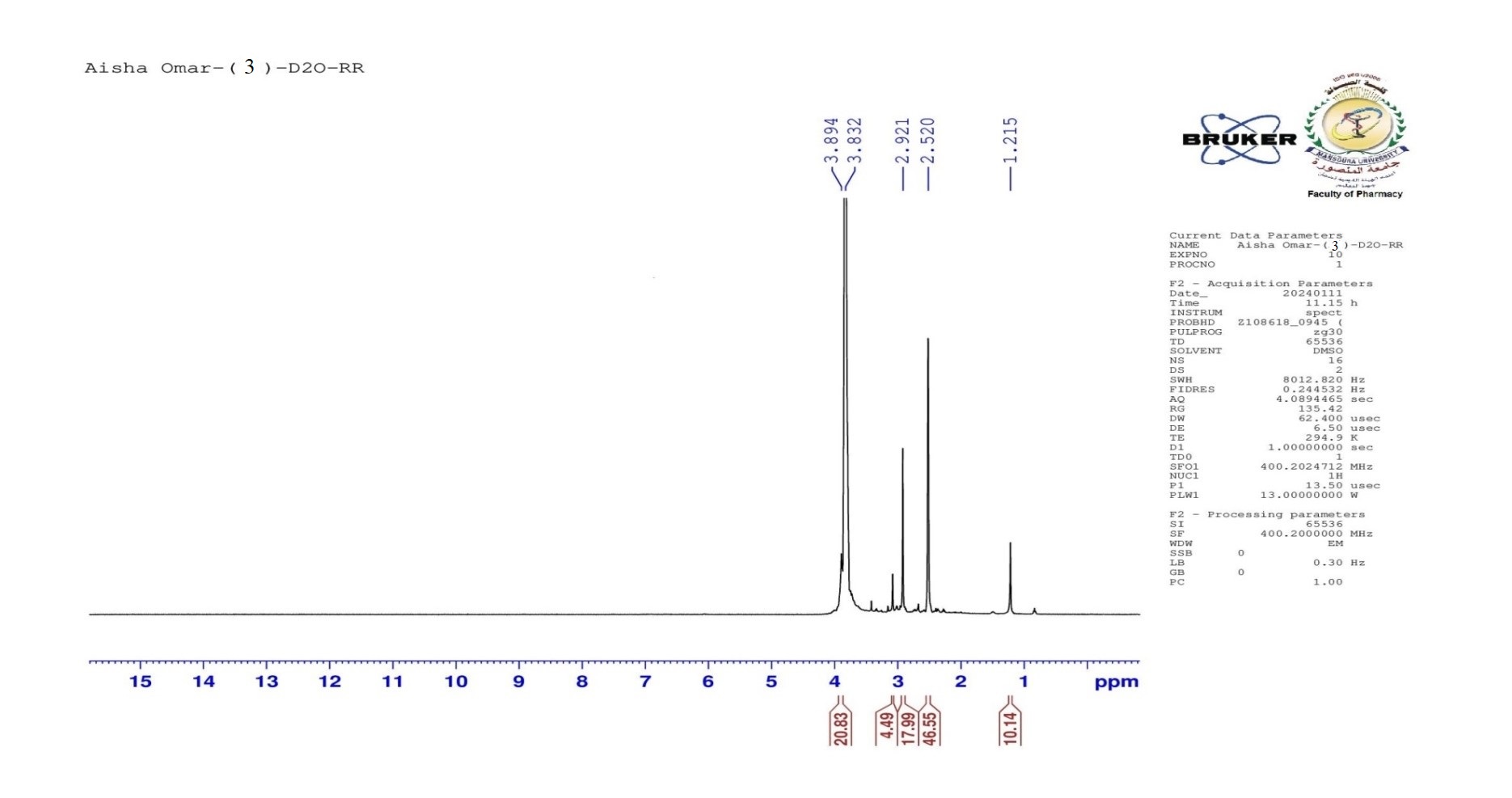


**Fig(S4)**: ^1^H NMR for compound **3**.


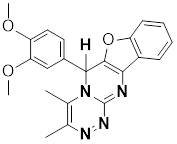

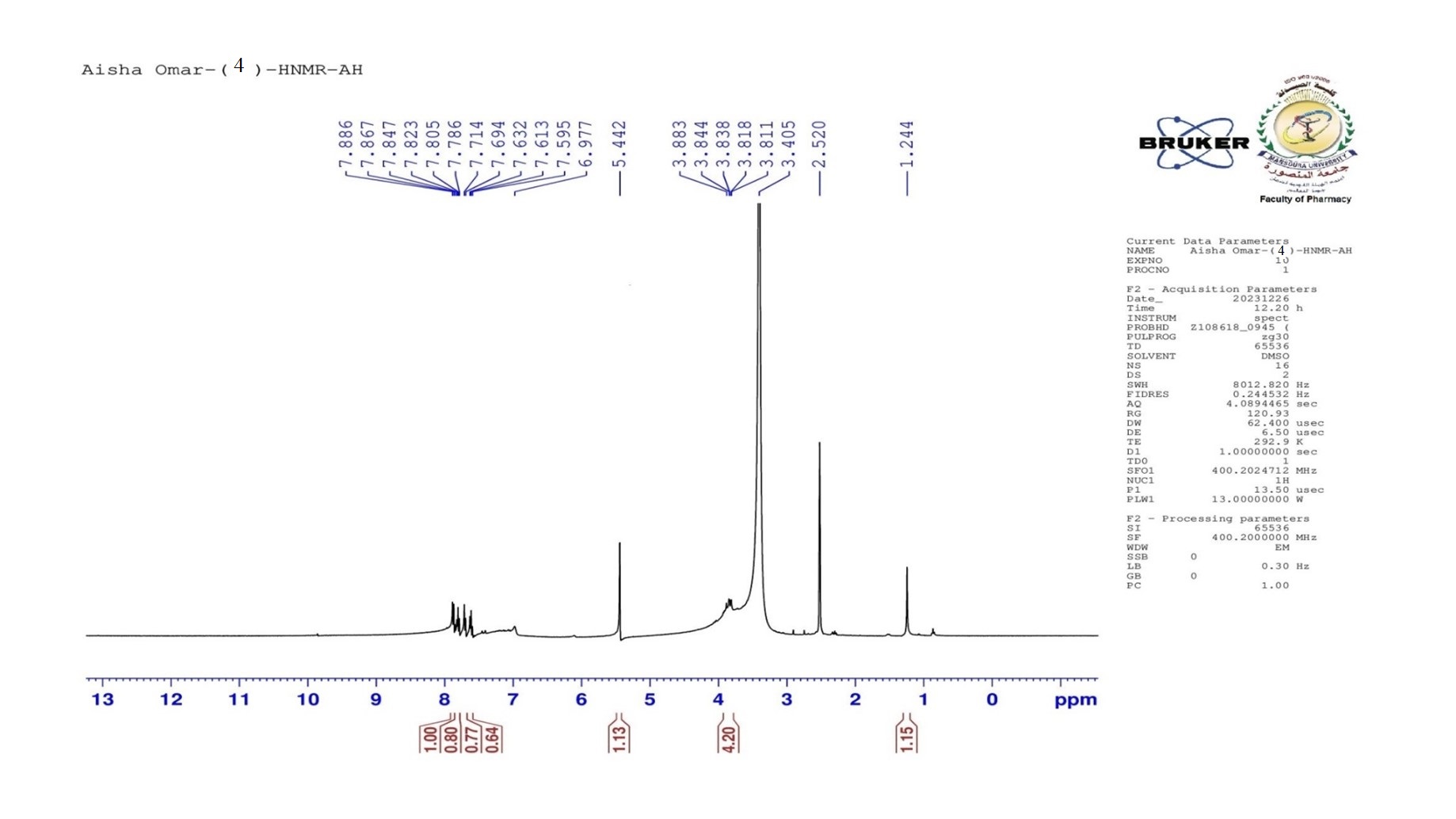


**Fig(S5)**: ^1^H NMR for compound **4**.


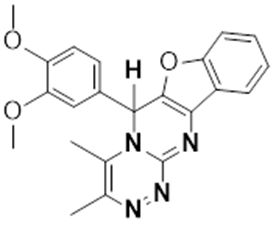

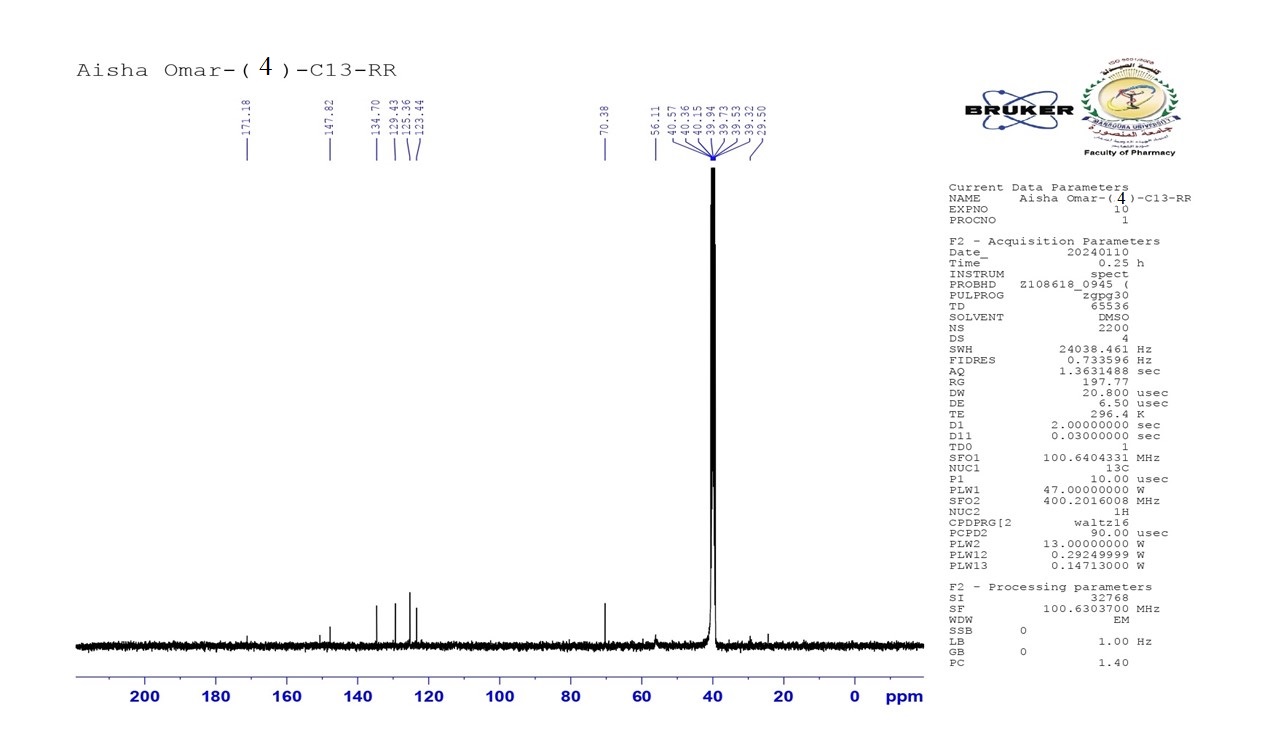


**Fig(S6)**: ^13^C NMR for compound **4**.


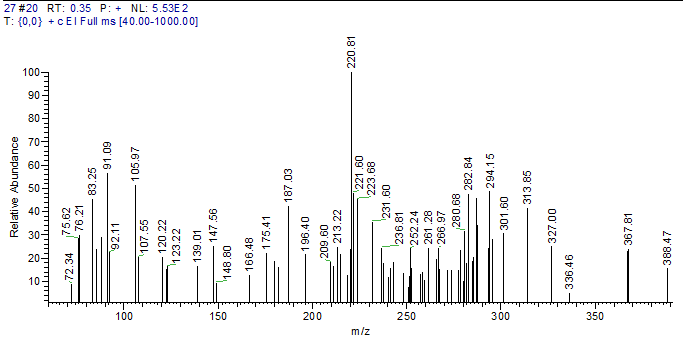

**Fig(S7)**: mass spectrum for compound **4**.

**
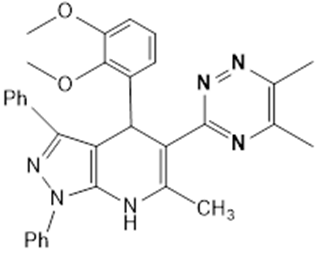

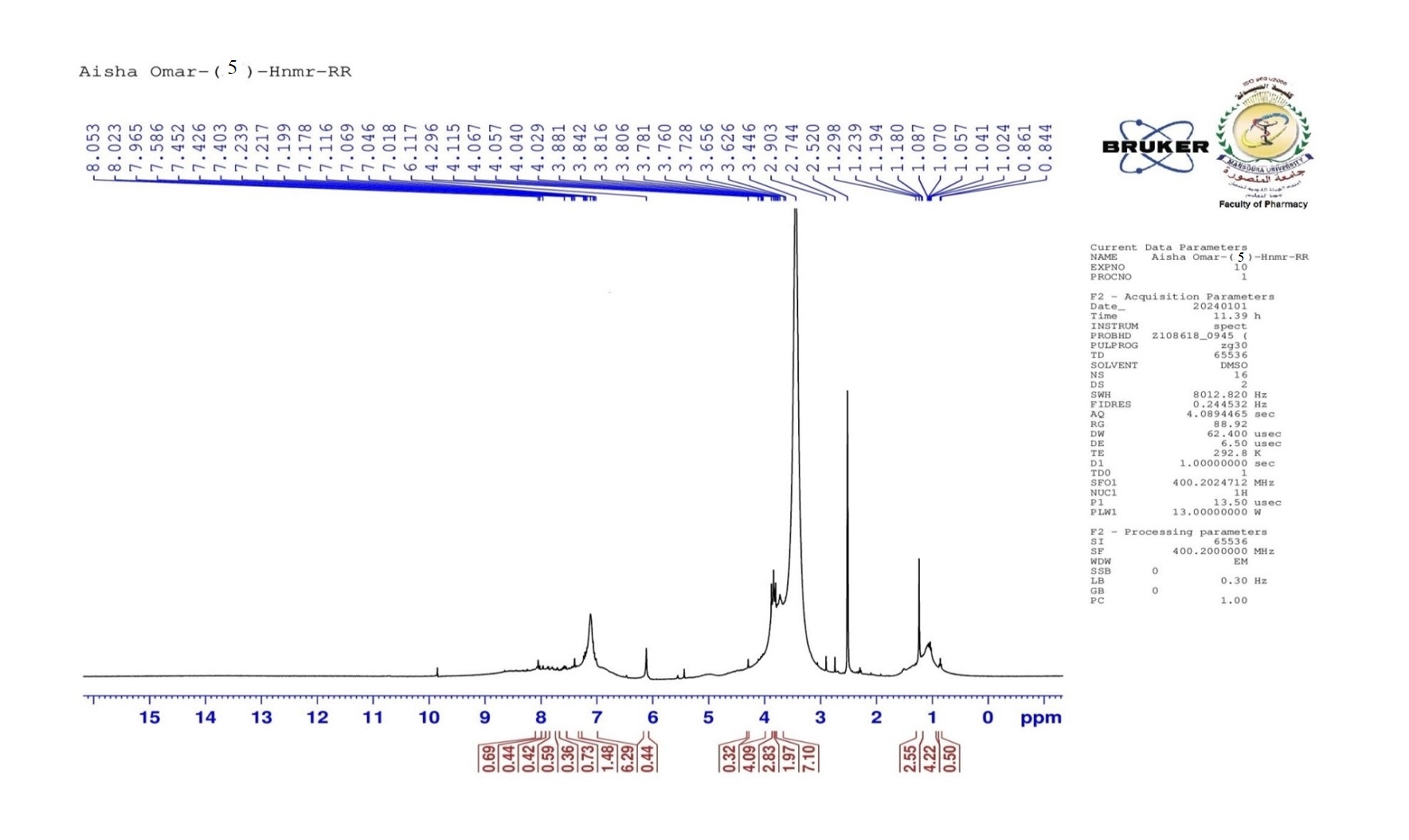
**

**Fig(S8):** ^1^H NMR for compound **5.**

**
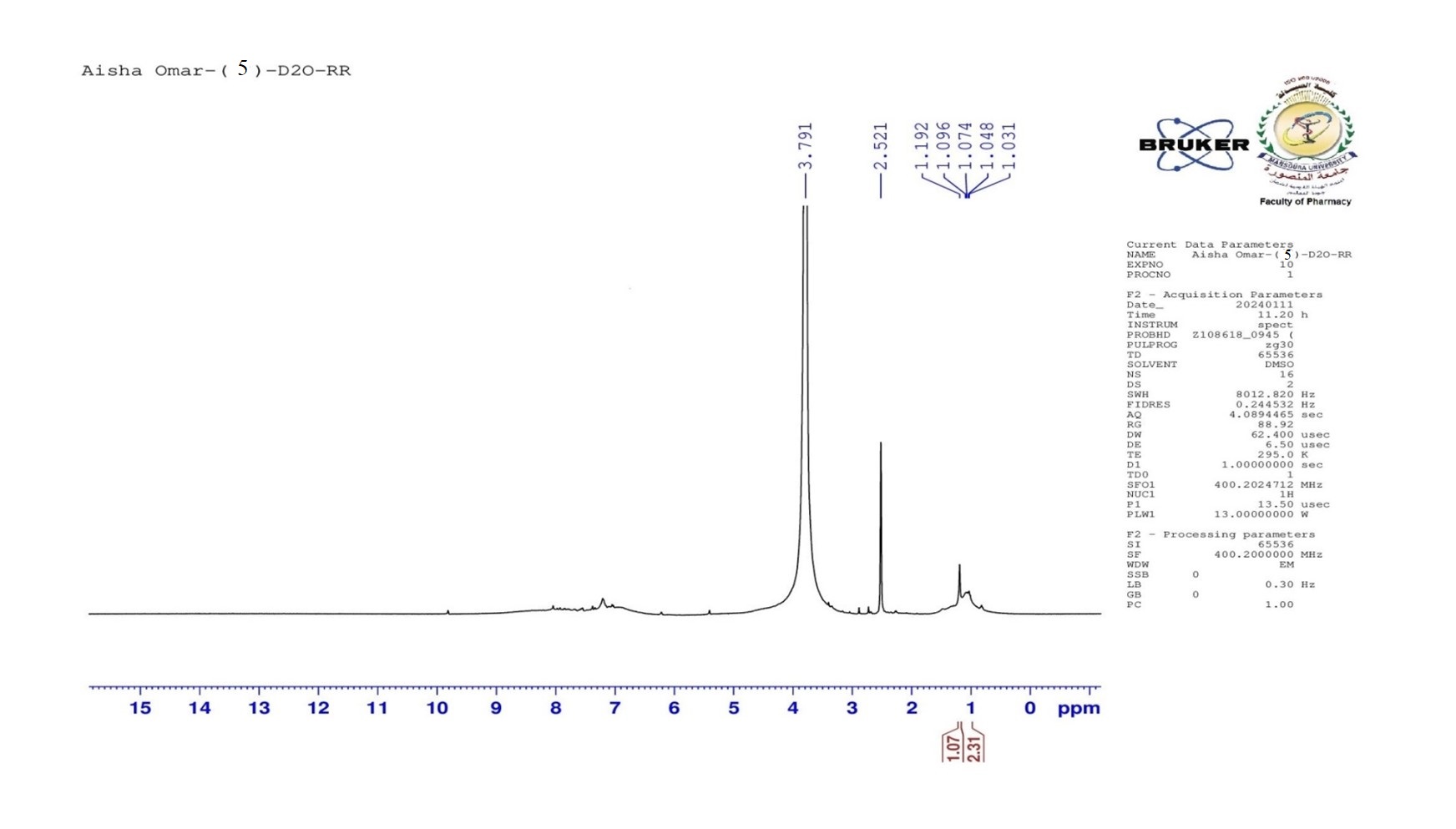
**

**Fig(S9):** D_2_O ^1^H NMR for compound **5.**


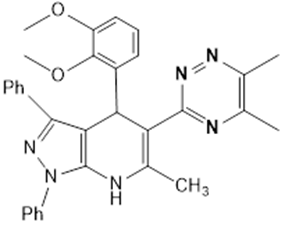

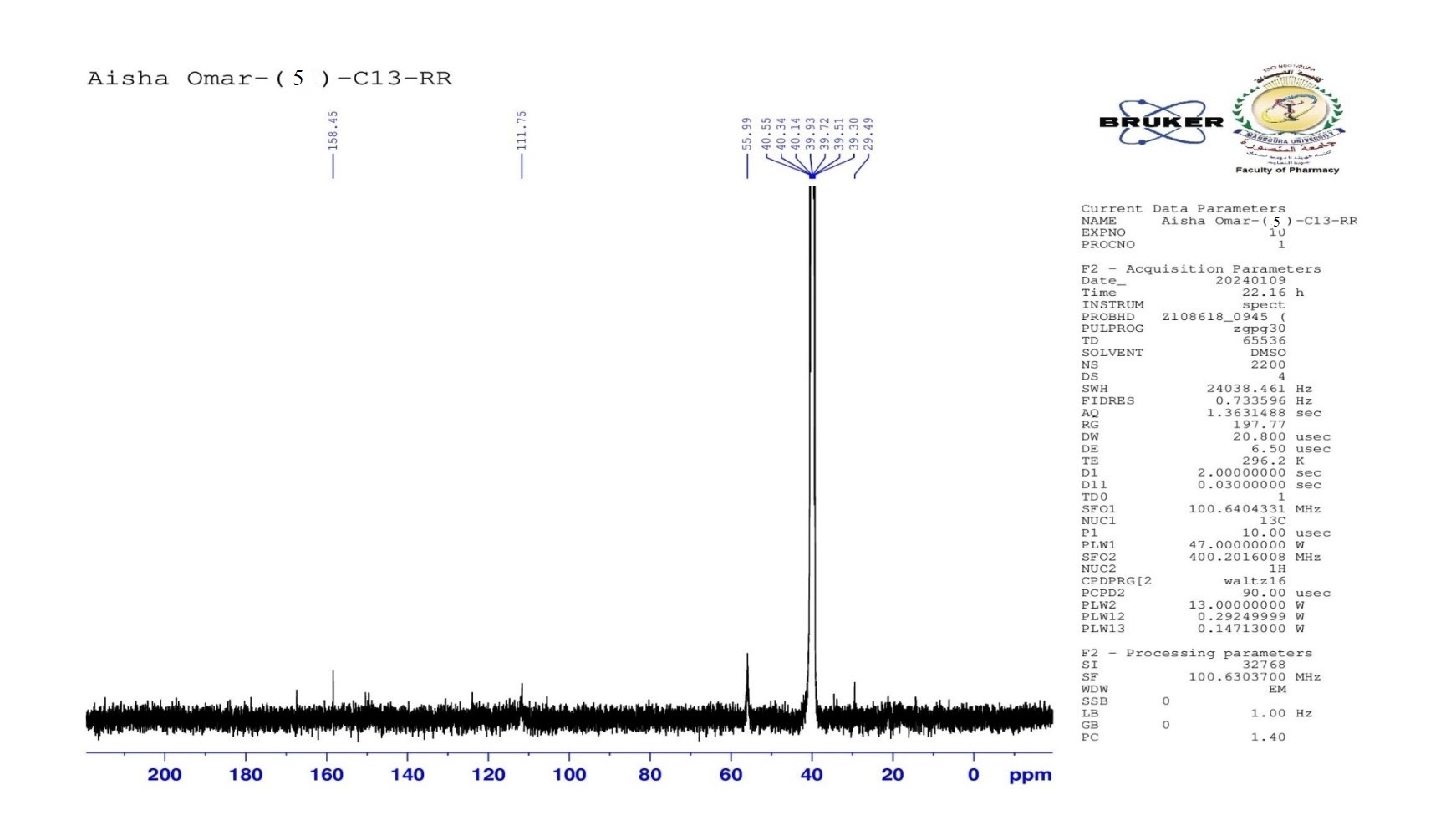


**Fig(S10):** ^13^C NMR for compound **5.**

**
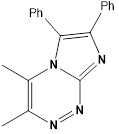

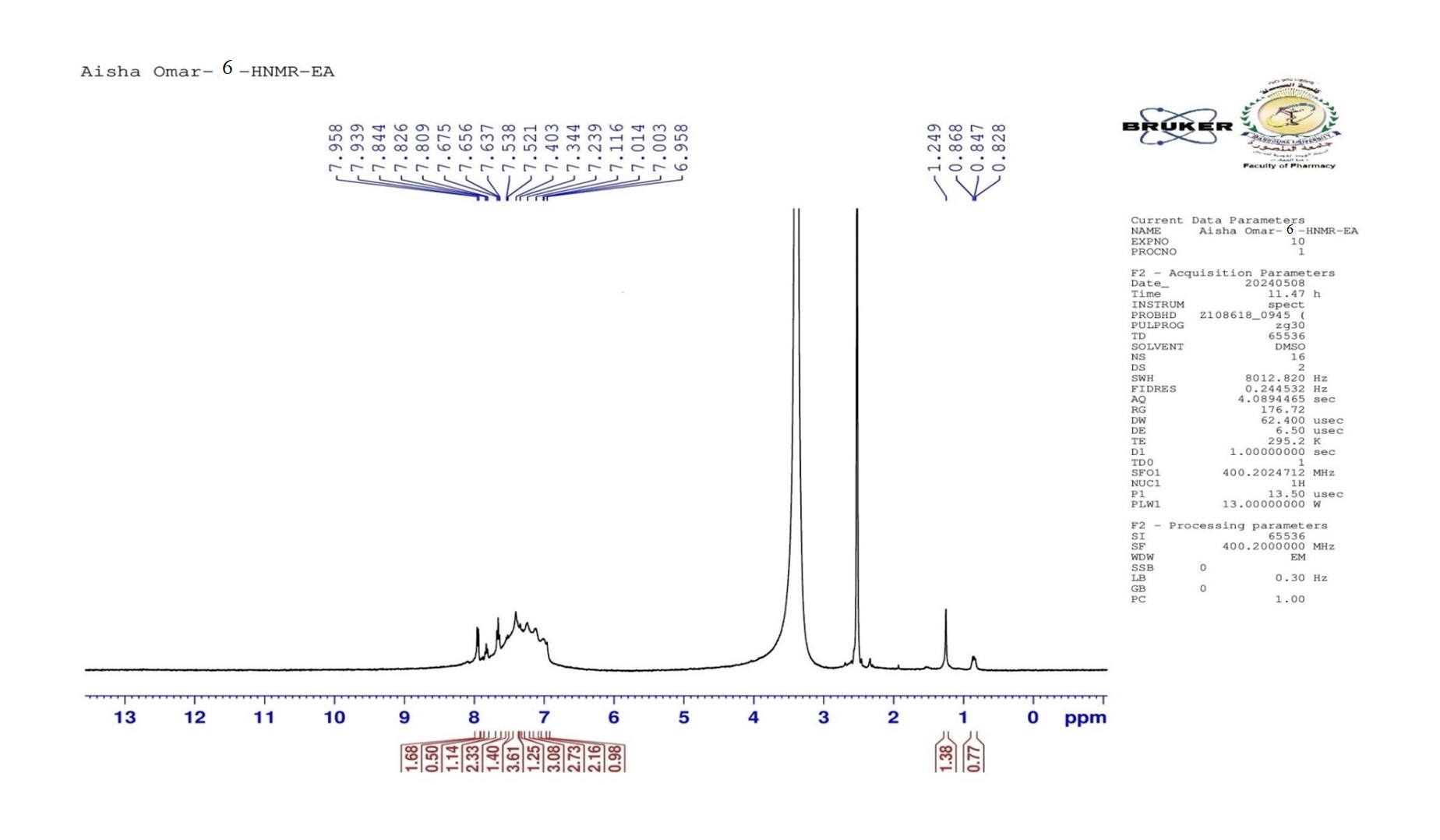
**

**Fig(S11):** ^1^H NMR for compound **6.**


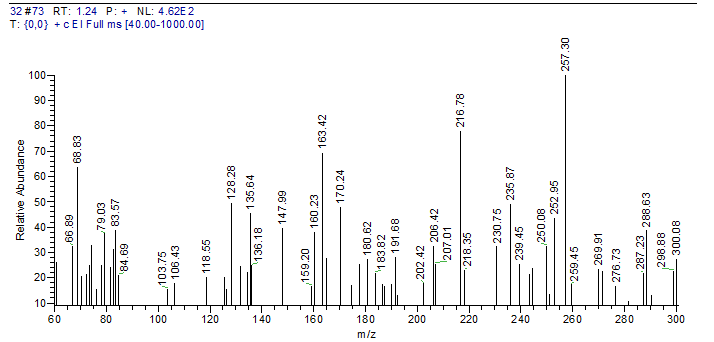

**Fig(S12)**: mass spectrum for compound **6.**


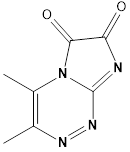

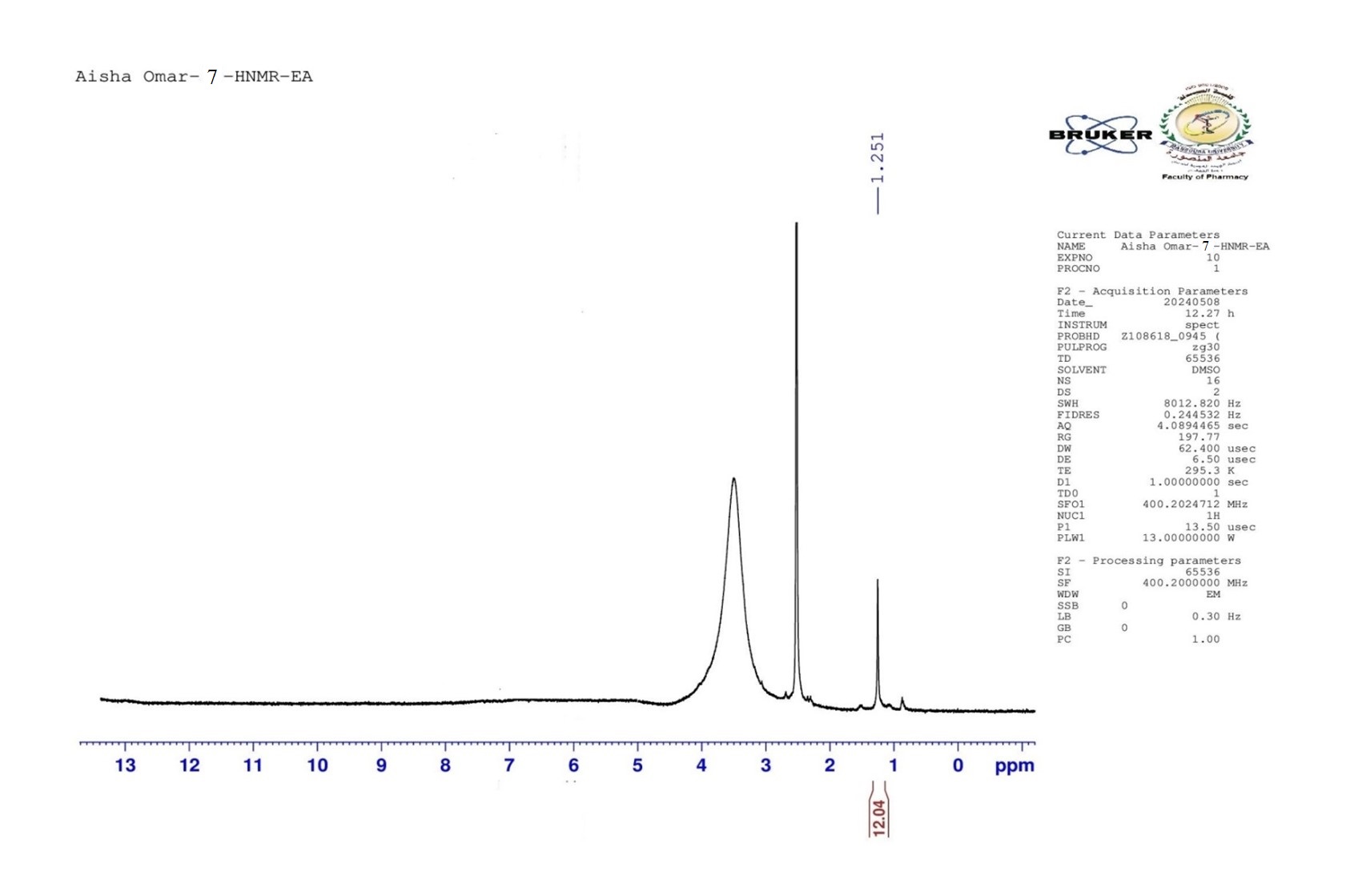


**Fig(S13):** ^1^H NMR for compound **7.**


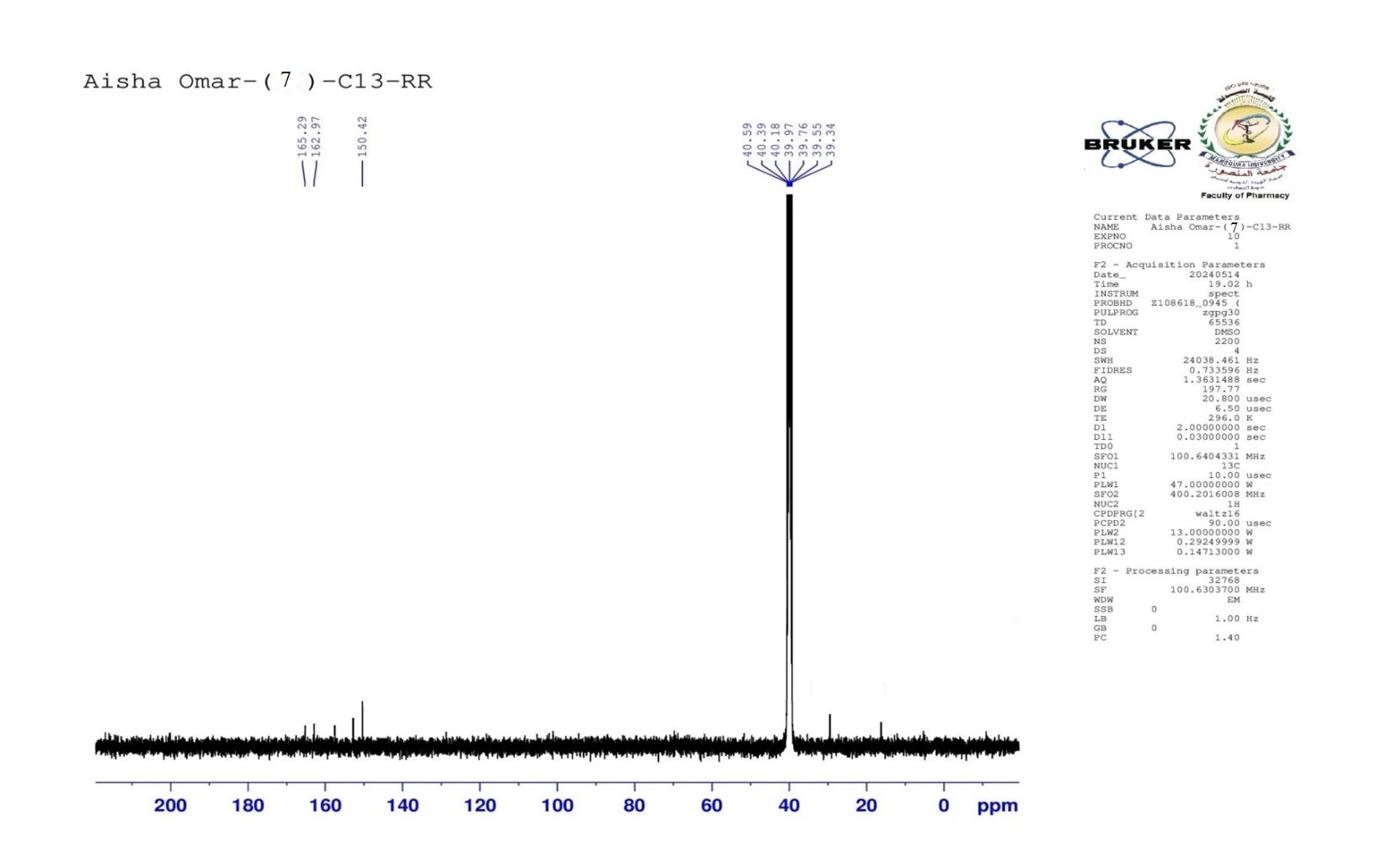

**Fig(S14):** ^13^CNMR for compound **7.**


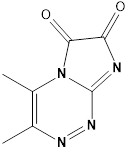

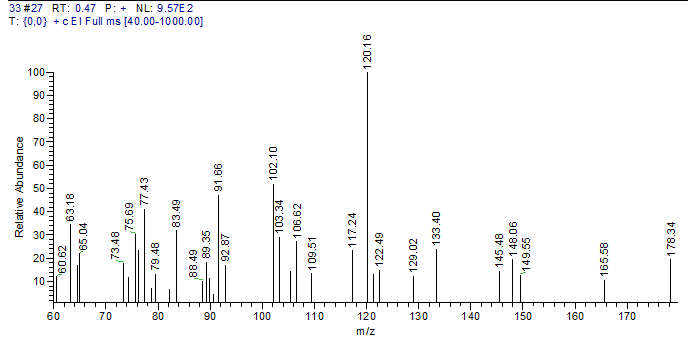


**Fig(S15)**: mass spectrum for compound **7.**

**
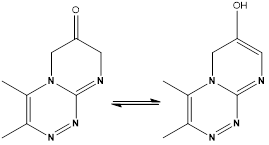

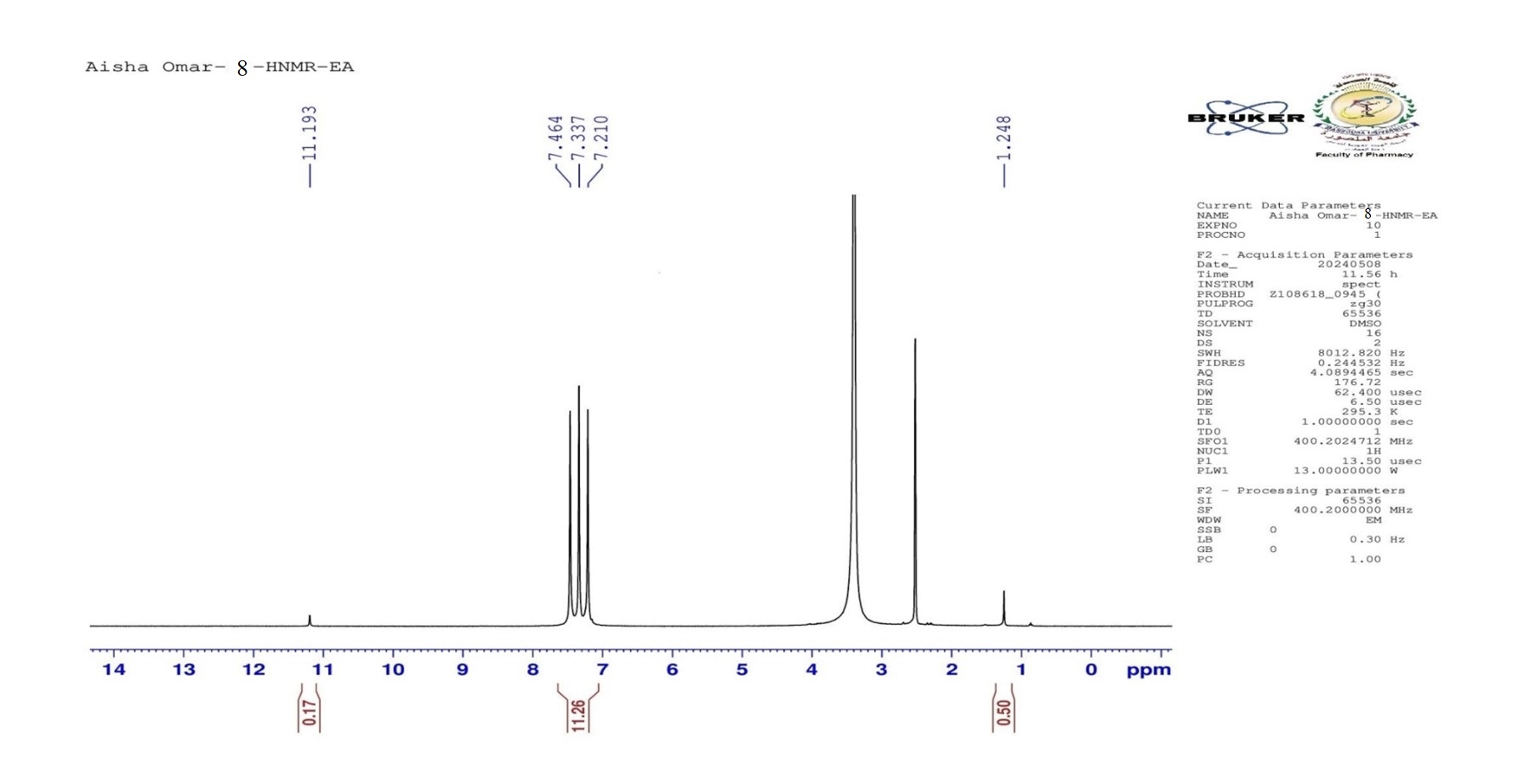
**

**Fig(S16):** ^1^H NMR for compound **8.**

**
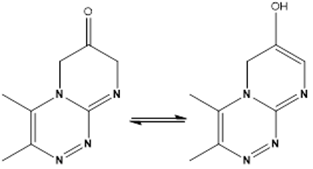

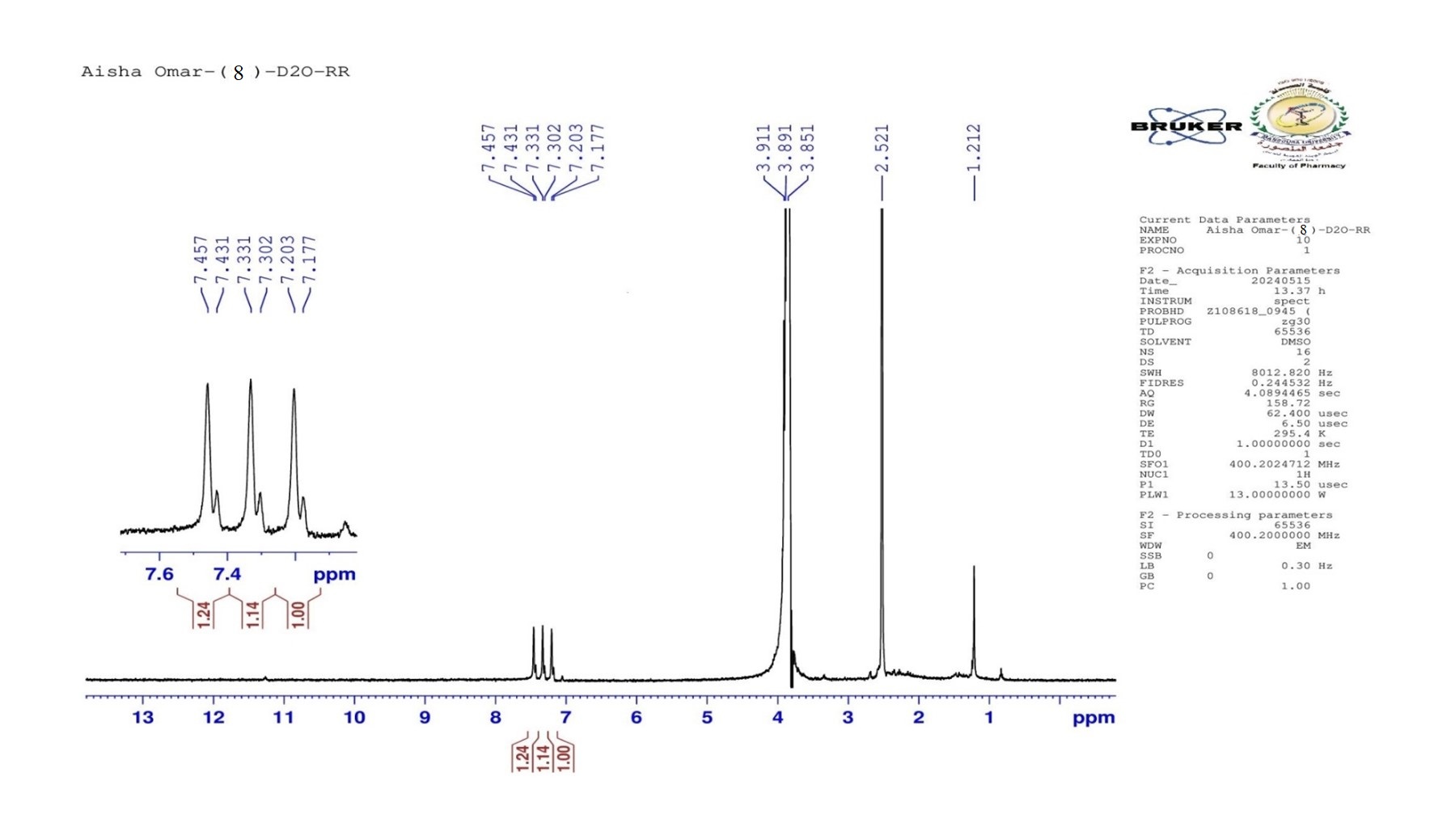
**

**Fig(S17):** D_2_O ^1^H NMR for compound **8.**

**
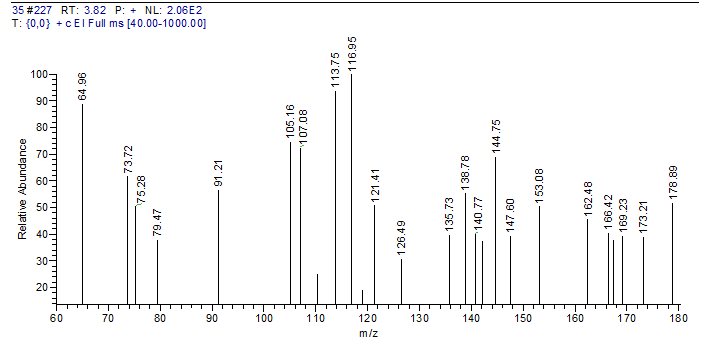
**

**Fig(S18)**: mass spectrum for compound **8.**

**
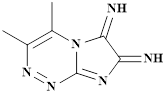
**
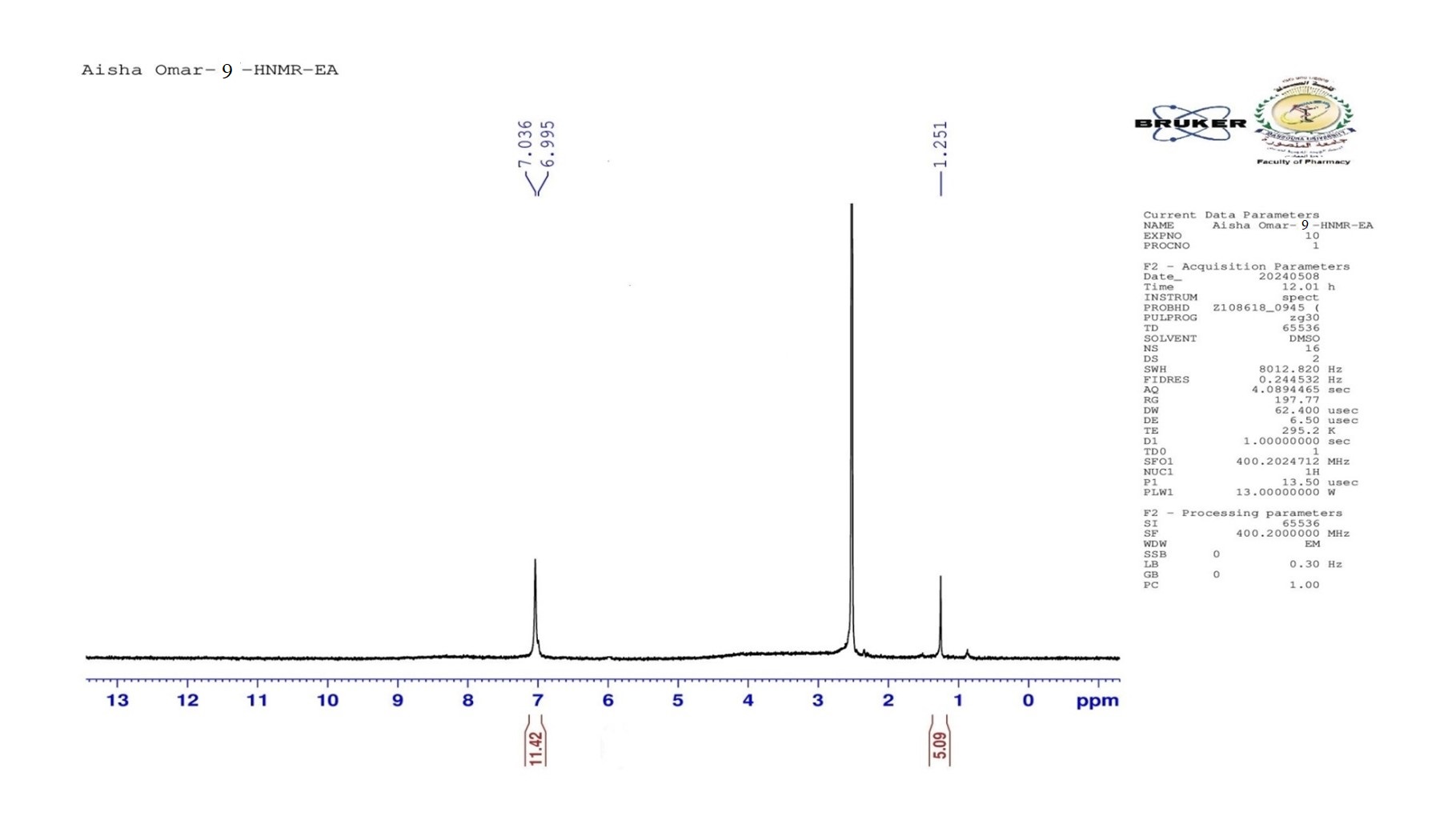


**Fig(S19):** ^1^H NMR for compound **9.**

**
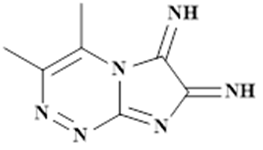

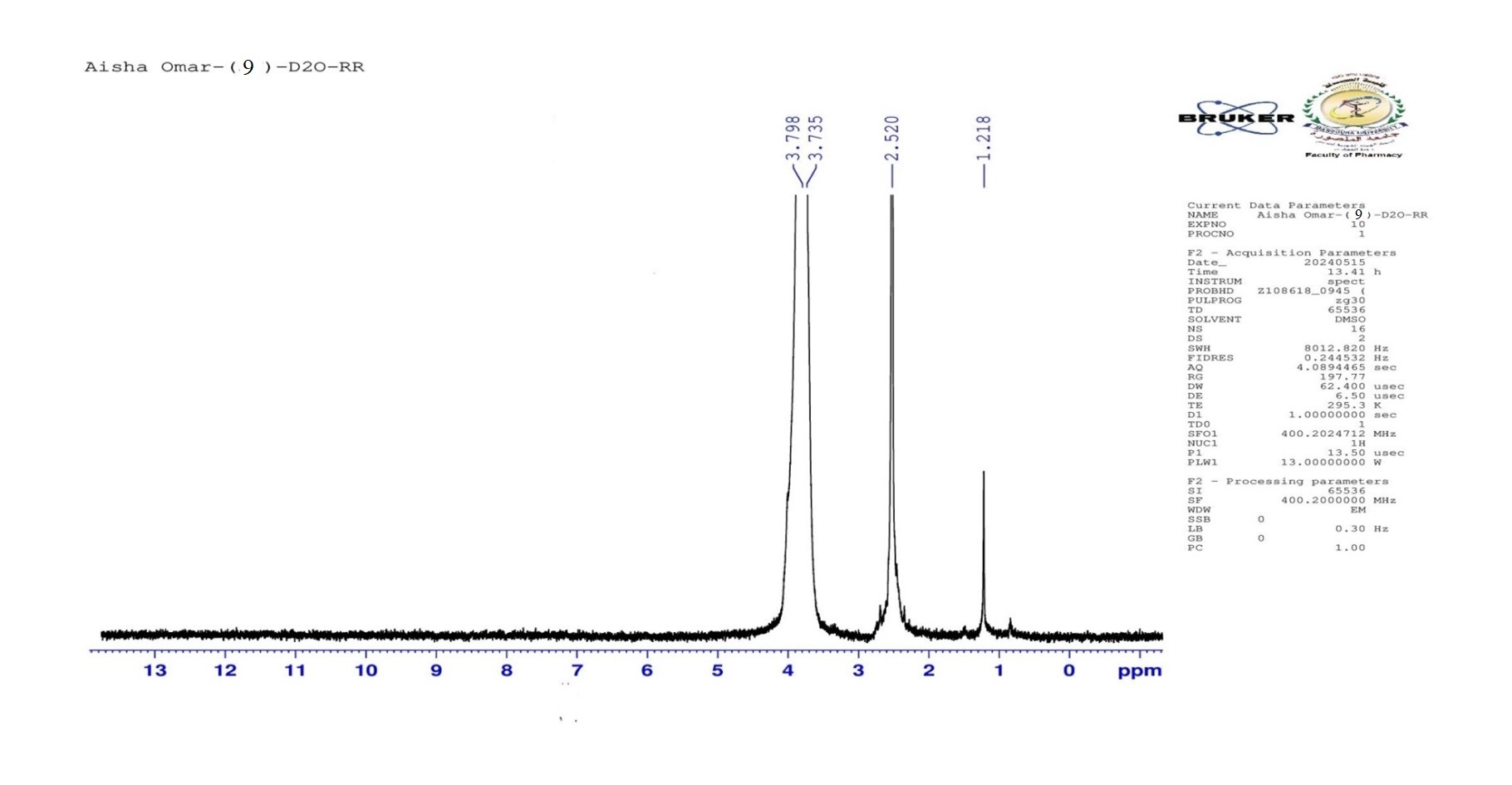
**

**Fig(S20):** D_2_O ^1^H NMR for compound **9.**

**
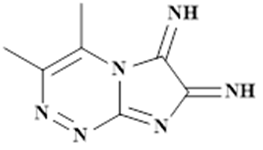

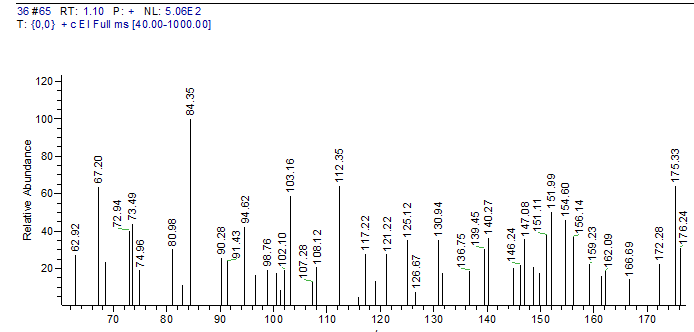
**

**Fig(S21)**: mass spectrum for compound **9.**

**
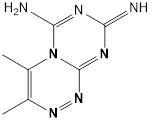

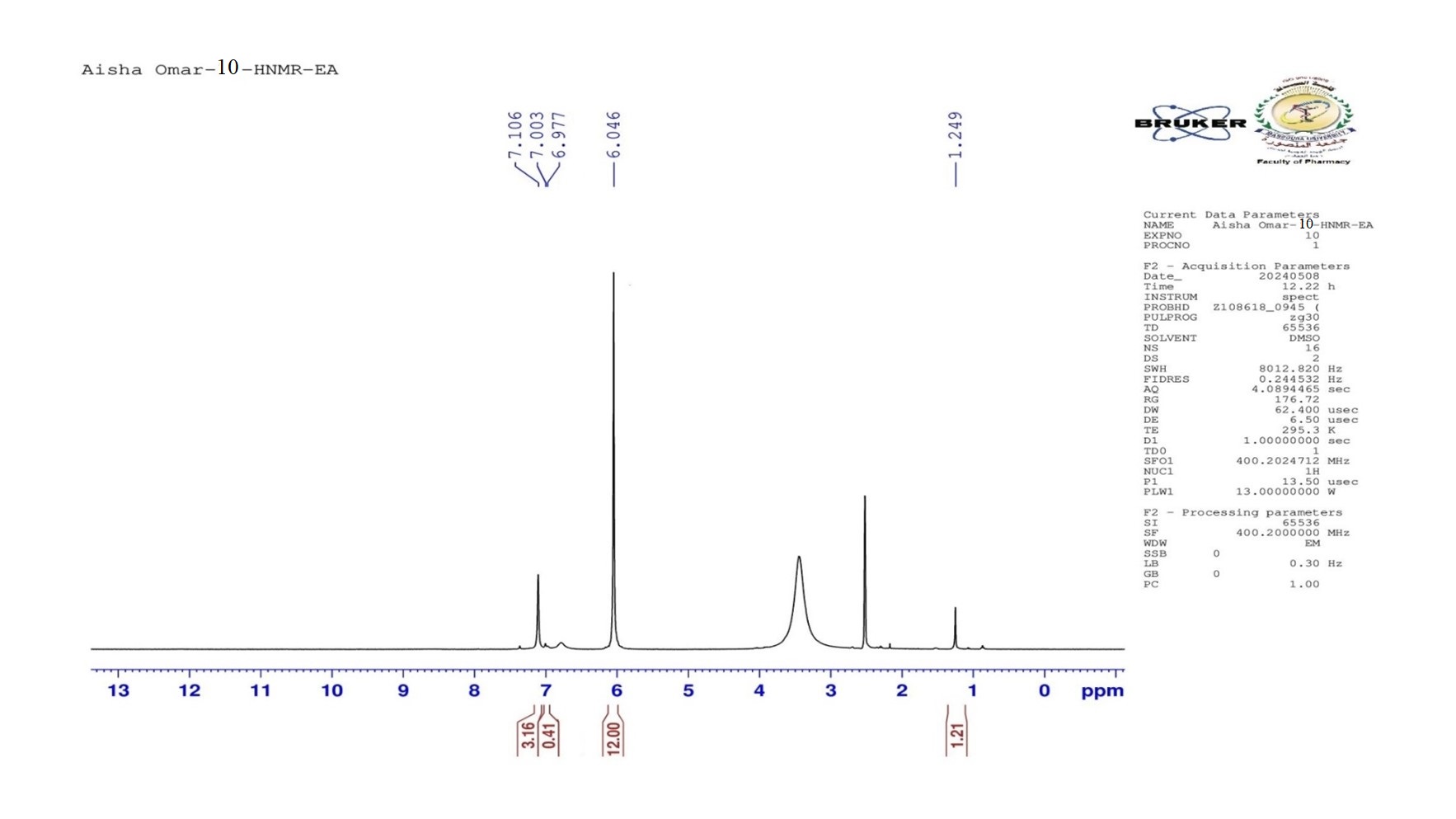
**

**Fig(S22):** ^1^H NMR for compound **10.**

**
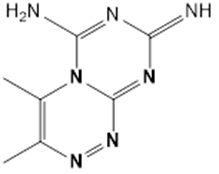

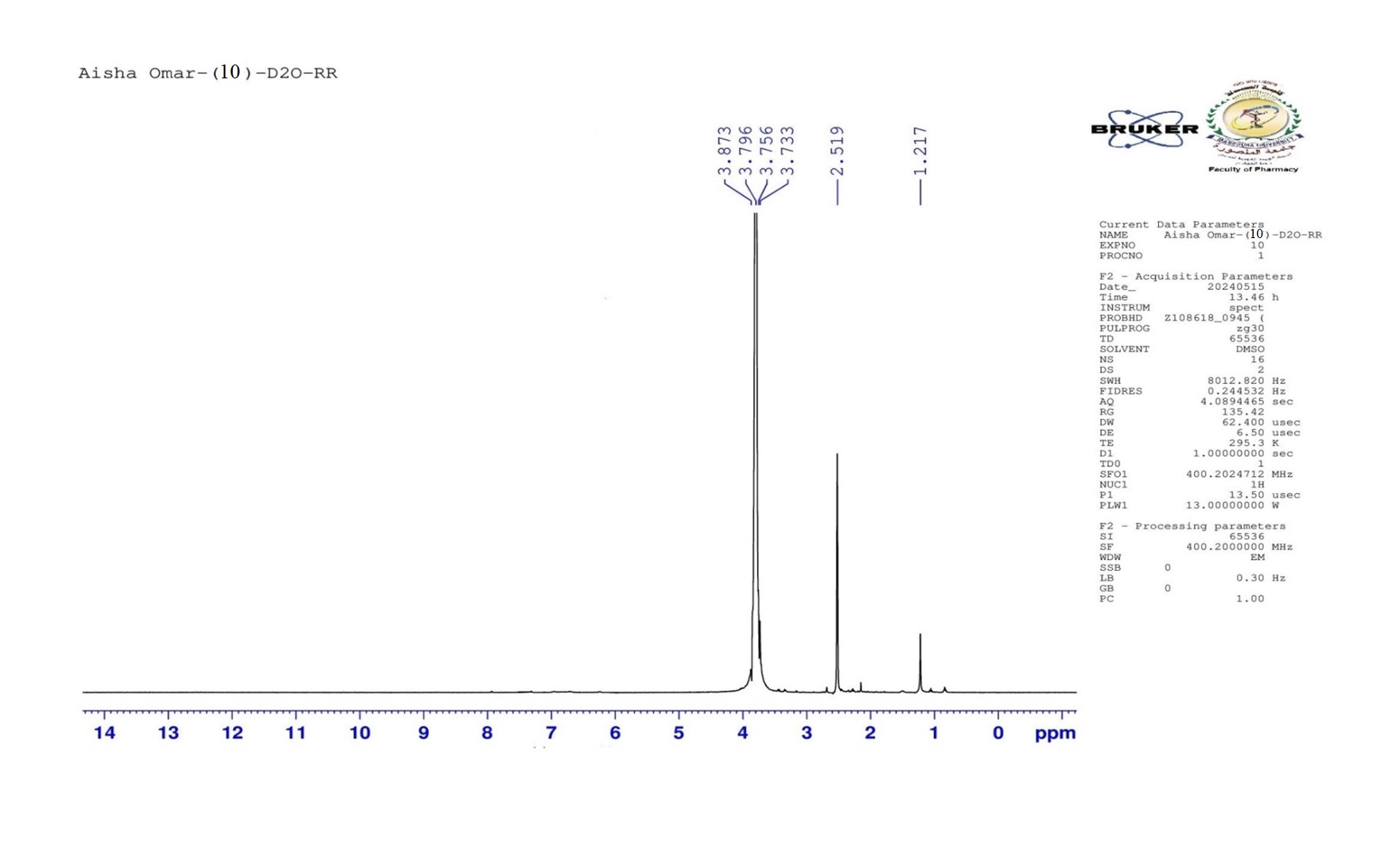
**

**Fig(S23):** D_2_O ^1^H NMR for compound **10.**

**
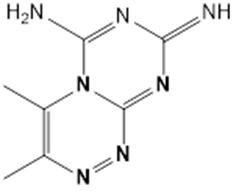

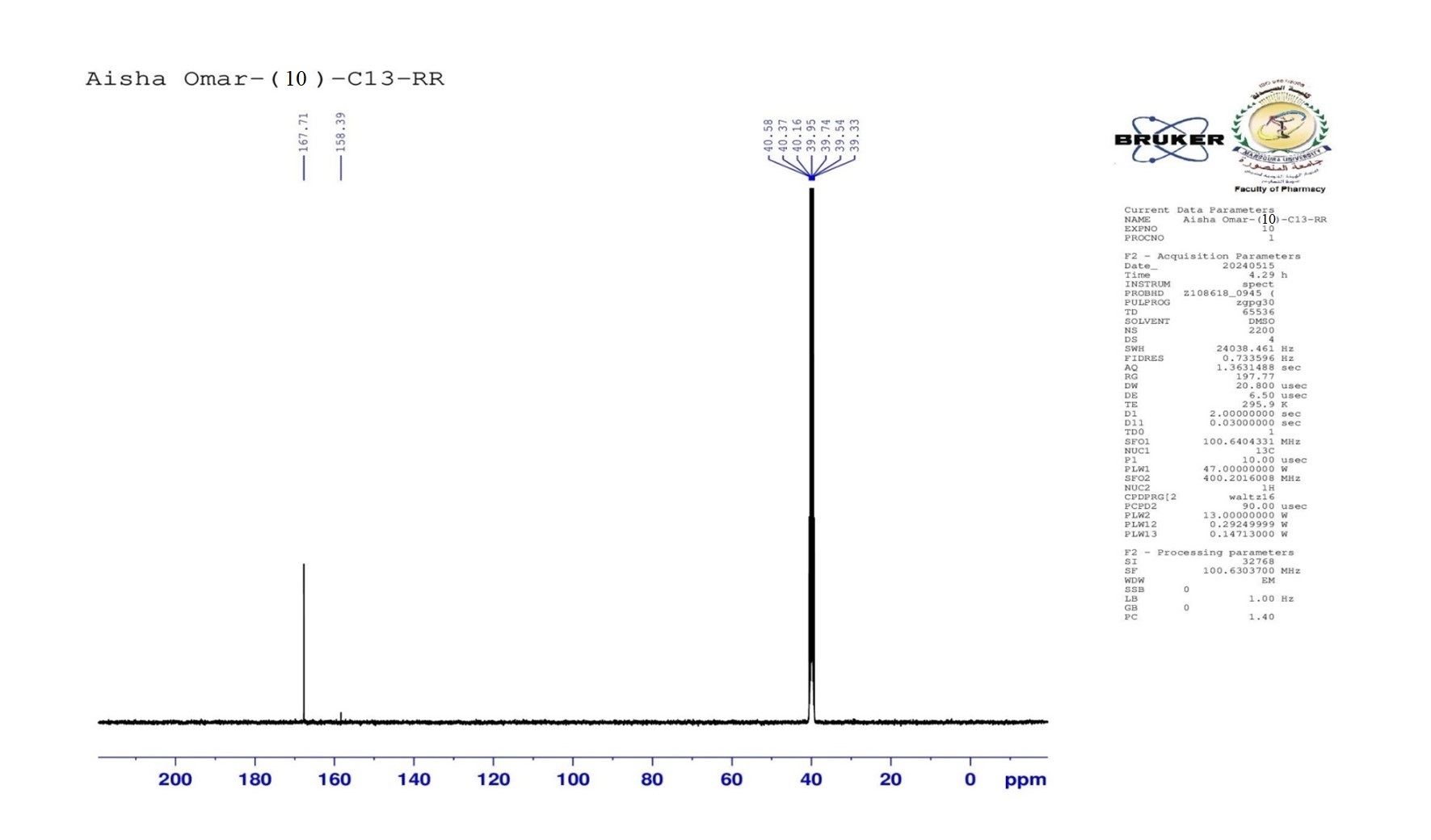
**

**Fig(S24):** ^13^C NMR for compound **10.**


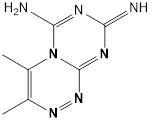

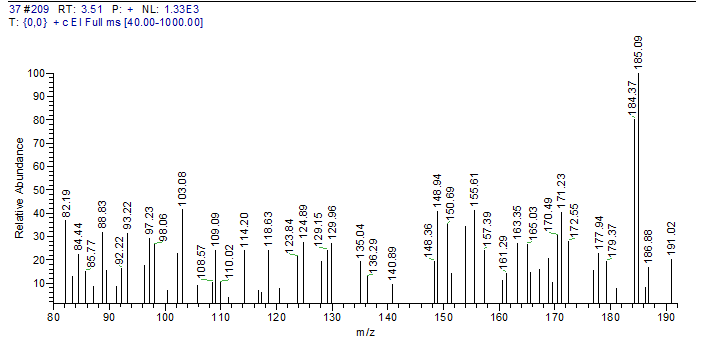


**Fig(S25)**: mass spectrum for compound **10.**

**
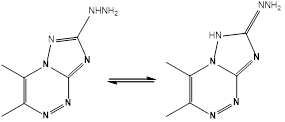

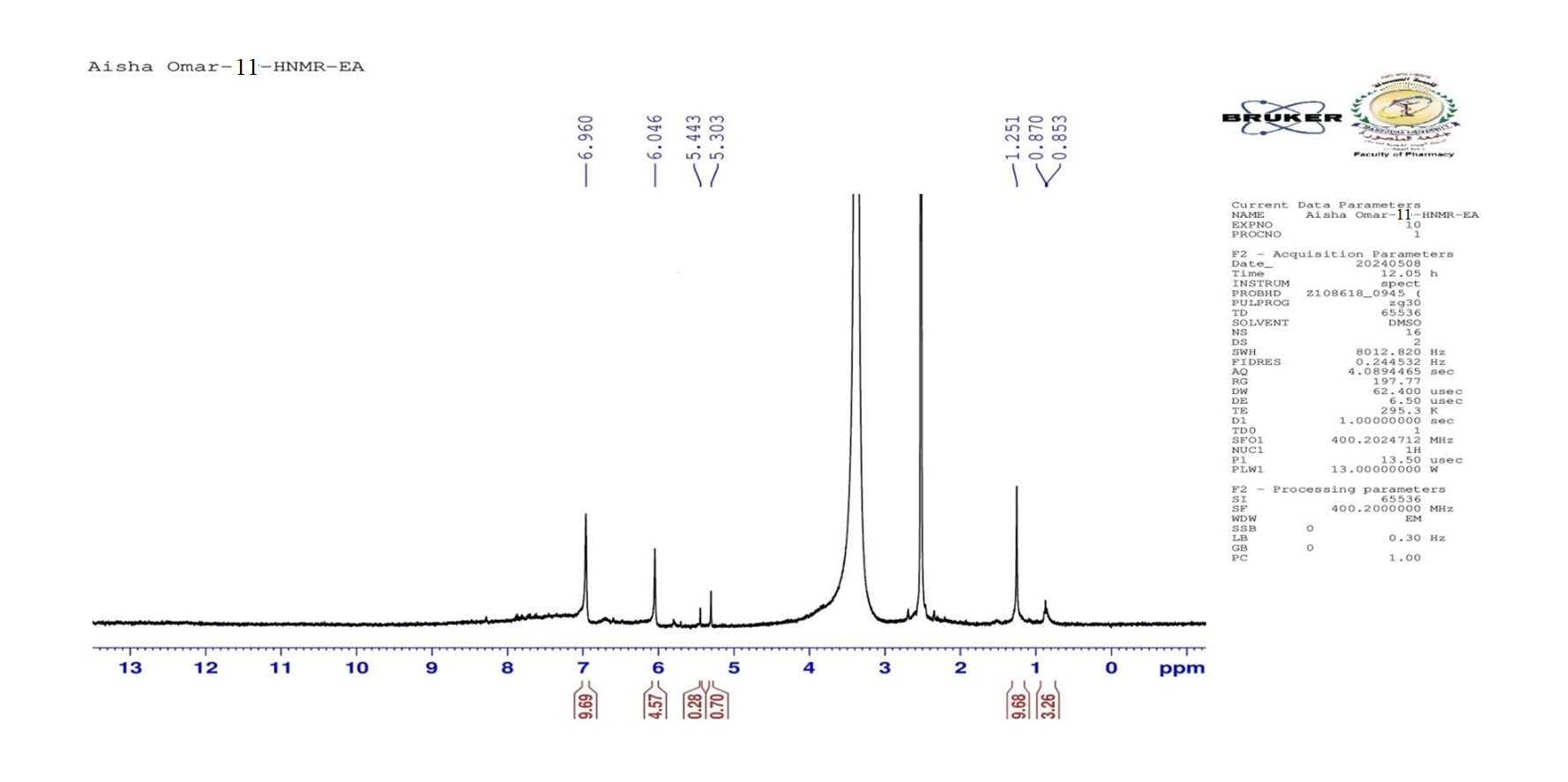
.**

**Fig(S26):** ^1^H NMR for compound **11.**

**
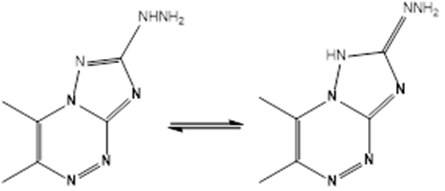

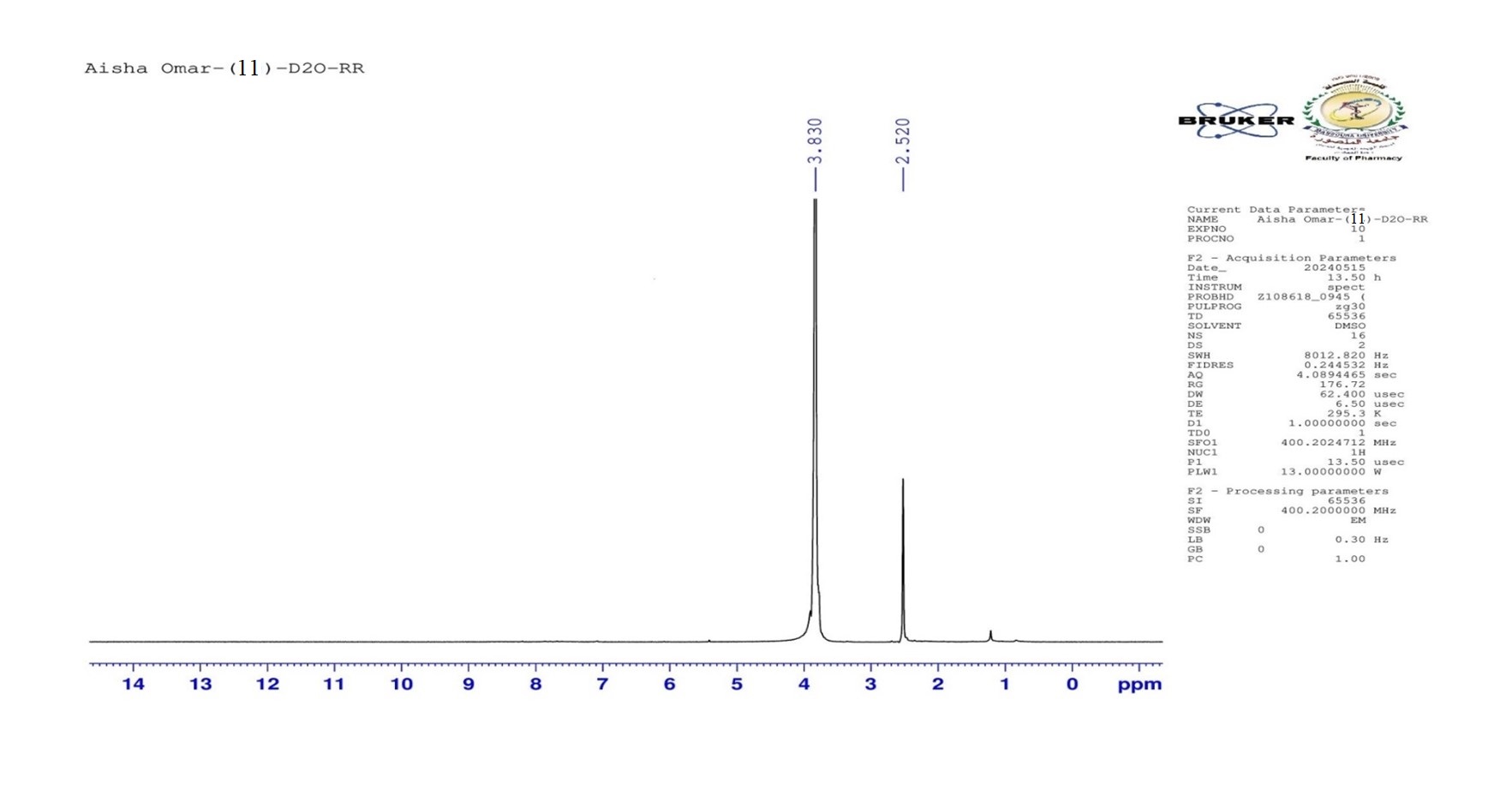
**

**Fig(S27):** D_2_O ^1^H NMR for compound **19.**

**
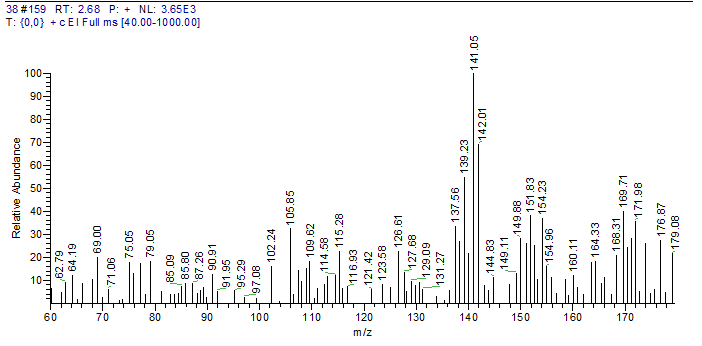
**

**Fig(S28)**: mass spectrum for compound **10.**

**Table (S1):** Docking interaction data calculations of compounds 2-4, 6-11, 13-16, 18, and19 inside 4KTNactive spots.

| **Compound** | **Binding affinity**  **(Kcal/mol)** | **Affinity Bond strength (Kcal/mol)** | **Affinity Bond length (in A^o^ from the main residue)** | **Amino acids** | **Ligand** | **Interaction** |
| --- | --- | --- | --- | --- | --- | --- |
|  |  | 4KTN |  |  |  |  |
| **2** | -6.33 | -0.4 | 3.79 | ASP 75 | Cl 15 | H-donor |
| **3** | -7.11 | -1.9  -7.6  -0.9  -0.7 | 2.99  2.93  3.61  3.66 | GLU 52  LYS 138  ARG 78  PRO 81 | N 11  O 15  N 24  6-ring | H-donor  H-acceptor  H-acceptor  pi-H |
| **4** | -6.91 | -0.9  -2.1 | 3.78  3.86 | ASN 48  ILE 80 | 5-ring  6-ring | pi-H  pi-H |
| **6** | -6.97 | -0.6 | 3.86 | ILE 80 | 6-ring | pi-H |
| **7** | -4.87 | -1.4  -0.6 | 2.94  3.62 | SER 122  ILE 80 | O 19  5-ring | H-acceptor  pi-H |
| **8** | -5.21 | -0.8  -0.6 | 2.89  3.47 | ILE 80  ASN 48 | O 23  6-ring | H-acceptor  pi-H |
| **10** | -5.30 | -1.1  -0.6 | 3.19  4.12 | SER 122  ASN 48 | N 9  6-ring | H-acceptor  pi-H |
| **11** | -5.36 | -1.1  -1.2 | 4.63  3.64 | ASN 48  ASN 48 | 5-ring  6-ring | pi-H  pi-H |

**Table (S2):** Docking interaction data calculations of compounds 2-4, 6-11, 13-16, 18, and19 inside 4WMZ active spots.

.

| **Compound** | **Binding affinity**  **(Kcal/mol)** | **Affinity Bond strength (Kcal/mol)** | **Affinity Bond length (in A^o^ from the main residue)** | **Amino acids** | **Ligand** | **Interaction** |
| --- | --- | --- | --- | --- | --- | --- |
| **4WMZ** | | | | | |  |
| **2** | -6.71 | -1.7  -0.6 | 3.63  4.10 | ARG 385  LEU 383 | N 20  6-ring | H-acceptor  H-pi |
| **3** | -7.36 | -0.8  -3.3 | 3.49  3.31 | HIS 468  LYS 151 | C 1  O 14 | H-donor  H-acceptor |
| **4** | -6.91 | -0.6  -0.6  -0.1 | 2.76  3.98  3.90 | PRO 462  THR 318  PHE 463 | C 40  5-ring  6-ring | H-donor  pi-H  pi-pi |
| **6** | -5.82 | -4.7  -2.5 | 3.13  3.32 | ARG 385  ARG 385 | N 3  N 4 | H-acceptor  H-acceptor |
| **7** | -4.74 | -1.0  -4.2 | 3.28  2.89 | ARG 385  ARG 385 | O 19  O 19 | H-acceptor  H-acceptor |
| **8** | -5.19 | -3.7  -5.0  -0.6  -0.6 | 3.02  3.27  3.77  3.72 | ARG 385  ARG 385  TYR 126  LEU 383 | N 4  N 7  C 15  6-ring | H-acceptor  H-acceptor  H-pi  pi-H |
| **10** | -5.27 | -0.7  -1.5  -0.7  -0.6 | 4.31  3.85  3.74  4.32 | CYS 470  CYS 470  LEU 380  LEU 383 | C 11  C 15  6-ring  6-ring | H-donor  H-donor  pi-H  pi-H |
| **11** | -5.00 | -1.3  -0.8  -1.4  -0.7  -0.9 | 3.60  3.02  3.13  3.72  4.11 | CYS 470  HIS 468  ARG 385  LEU 380  PHE 463 | C 11  N 20  N 20  6-ring  6-ring | H-donor  H-donor  H-acceptor  pi-H  pi-H |
